# CARD16 restores tumorigenesis and restraints apoptosis in glioma cells Via FOXO1/TRAIL axis

**DOI:** 10.1038/s41419-024-07196-2

**Published:** 2024-11-08

**Authors:** Ruoheng Xuan, Tianyu Hu, Lingshan Cai, Beichuan Zhao, Erqiao Han, Zhibo Xia

**Affiliations:** Department of Neurosurgery, The First Affiliated Hospital of Sun Yat-sen University, Guangzhou, China

**Keywords:** CNS cancer, Prognostic markers, Apoptosis

## Abstract

A hallmark of glioma cells, particularly glioblastoma multiforme (GBM) cells, is their resistance to apoptosis. Accumulating evidences has demonstrated that CARD16, a caspase recruitment domain (CARD) only protein, enhances both anti-apoptotic and tumorigenic properties. Nevertheless, there is a limited understanding of the expression and functional role of CARD16 in glioma. This study seeks to investigate, through in silico analysis and clinical specimens, the role of CARD16 as a potential tumor promoter in glioma. Functional assays and molecular studies revealed that CARD16 promotes tumorigenesis and suppresses apoptosis in glioma cells. Moreover, knockdown of CARD16 enhances the expression of the FOXO1/TRAIL axis in GBM cells. Additionally, FOXO1 downregulation in CARD16 knockdown GBM cells restores proliferation and reduces apoptosis. Further investigation demonstrated that elevated P21 expression inhibits CDK2-mediated FOXO1 phosphorylation and ubiquitination in CARD16-knockdown GBM cells. Collectively, these findings suggest that CARD16 is a tumor-promoting molecular in glioma via downregulating FOXO1/TRAIL axis, and suppressing TRAIL-induced apoptosis. The CARD16 gene presents significant potential for prognostic prediction and advances in innovative apoptotic therapeutics.

## Introduction

Despite intensive therapeutic interventions such as surgical resection, radiation, and chemotherapy, glioblastoma (GBM) remains as the most aggressive form of glioma in adults [1], exhibiting a five-year survival rate of merely 5.8% [2]. Apoptosis hinges on the activation of apoptotic signaling cascades [3, 4], which are often disrupted in malignancies [5]. Currently, there are few therapies that effectively induce apoptosis in GBM cells, beyond the standard treatment [6, 7].

TNF-related apoptosis-inducing ligand (TRAIL) plays a pivotal role in the extrinsic apoptotic pathway [8]. Initial investigations have demonstrated that TRAIL and Death receptor 5 (DR5) elicit apoptosis in glioma, thereby improving patient outcomes [9, 10]. Nevertheless, GBM tumors predominantly exhibit resistance to TRAIL-induced apoptosis [11]. Forkhead Box O (FOXO) transcription factors are situated upstream of the FOXO pathway involving TRAIL-DR5 [12–16]. Notably, FOXO1 functions as a critical tumor suppressor by activating cell cycle arrest and apoptosis in glioma [17–19]. However, the exact mechanism by which glioma downregulates FOXO1 remains unclear.

Caspase Recruitment Domain Family Member 16 (CARD16) facilitates potential anti-apoptotic and tumorigenic capabilities through its binding affinity to CARD domains [20]. Previous research has suggested that CARD16 may play a role in the preservation of stemness in GBM [21]. However, our current knowledge understanding of CARD16 expression and functionality in glioma remains limited. Here, we present findings that CARD16 plays a pivotal role in proliferation and anti-apoptosis by downregulating the FOXO1/TRAIL pathway in glioma.

## Results

### CARD16 expression is upregulated in glioma and correlated with prognosis

**Fig. 1:**
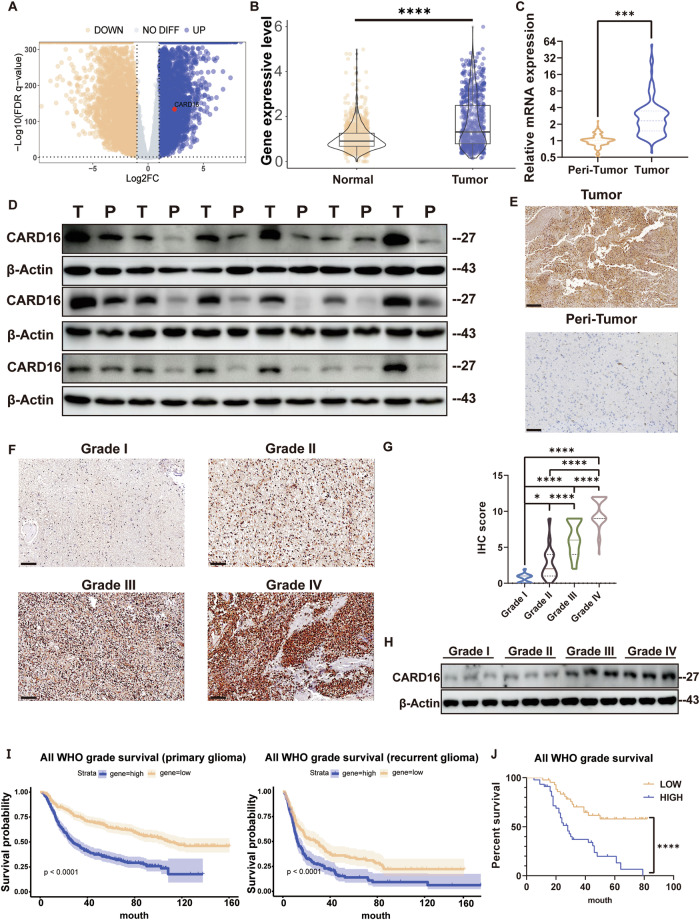
CARD16 expression is upregulated in glioma and associated with prognosis. **A** Volcano plot of up-regulated and down-regulated genes in TCGA and GTEx. X-axis: log2 ratio of RNA expression levels between normal and tumor tissues. Y-axis: the FDR q-value (−log10 transformed) of RNAs. The red dot indicates gene expression of CARD16. **B** Gene expression level was compared between glioma samples(*n* = 697) and normal brain tissues (*n* = 1153) genes in TCGA and GTEx database (*P* < 0.0001). **C**–**E** CARD16 expression level of paired tumor and peri-tumor tissue samples were detected by **C** qPCR (*n* = 40, *P* < 0.001), **D** WB(*n* = 40) and **E** IHC(*n* = 30). Scale bar, 100 μm. **F, G** Representative IHC images and IHC scores of CARD16 expression level in glioma samples(*n* = 100). Scale bar, 100 μm. **H** WB assay of CARD16 expression level in glioma samples of different WHO grades. **I** Log-rank analysis of CARD16 expression in GBM of CGGA dataset. Left, OS of primary glioma patients. Right, OS of recurrent glioma patients. **J** Survival analysis of glioma patients based on CARD16 expression. With an IHC-Score >7 being defined as high expression and an IHC-Score ≤7 being defined as low expression. Statistical results are presented as mean ± SD, *p < 0.05, **p < 0.01, ***p < 0.001, ****p < 0.0001. Data are representative from at least three experiments with similar results. Error bars represent independent experiments.

To identify the differential expression genes (DEGs) between GBM and normal brain samples, an analysis was conducted using the TCGA and GTEx databases (Fig. 1A). Among the top 300 DEGs, eleven genes overlapped with survival-related gene in GBM (Supplementary Fig. S1A, B, C). Of these genes, CARD16 has been previously documented to impede the differentiation of GSCs [21]. Furthermore, elevated levels of CARD16 expression were observed in GBM compared to normal tissues, as demonstrated in both database (Fig. 1B) and clinical specimens (Fig. 1C, D, E).

To further elucidate the impact of CARD16 on prognosis, IHC assays were conducted on glioma specimen slides. The results indicated that a significant increase in IHC scores with the progression of WHO grades (Fig. 1F, G). This trend was also corroborated by WB assay (Fig. 1H). Additionally, we re-interrogated the overall survival (OS) data in the TCGA and CGGA database. Our findings revealed that patients with higher CARD16 expression exhibited significantly shorter OS (Fig. 1I, Supplementary Fig. S1D). A cut-off value of seven was designated for the IHC-Score of glioma slides. The comparison of OS between CARD16 high and low expression groups demonstrated that higher CARD16 expression exhibited a poorer prognosis (Fig. 1J). Briefly, CARD16 expression was upregulated in glioma and exhibited a negative correlation with patient prognosis.

### CARD16 is overexpressed in GBM cell lines and associated with tumorigenesis

**Fig. 2:**
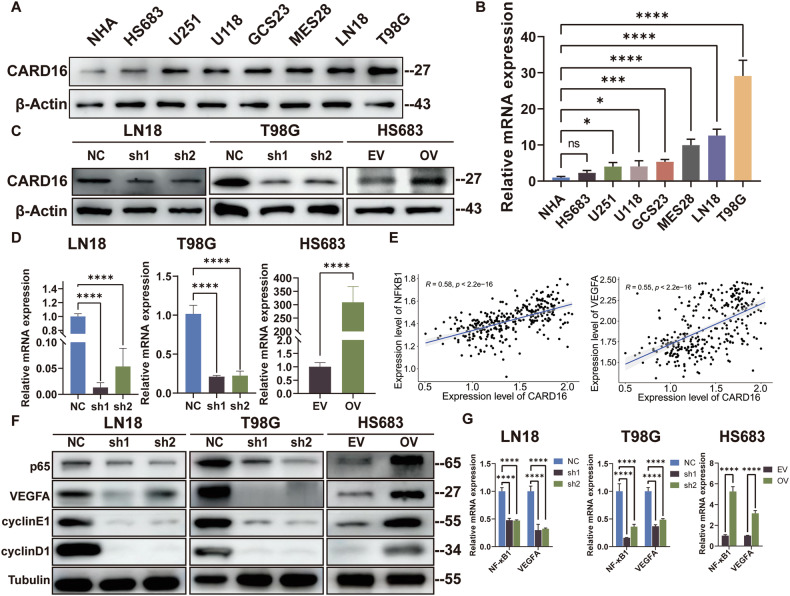
CARD16 is upregulated in GBM cell lines and associated with tumorigenesis. **A** WB of NHA, GBM cell lines, and GSCs using antiCARD16 antibodies. **B** CARD16 mRNA expression level in NHA, GBM cell lines, and GSCs. **C, D** WB and qRT-PCR of CARD16 expression in CARD16 knockdown GBM cell lines LN18 and T98G and CARD16-overexpressing glioma cell line HS683. **E** Correlation of CARD16 and NFKB1 and VEGFA expression in CGGA databases. **F, G** Tumor proliferation marker NFKB1-p65, tumor angiogenesis marker VEGFA, and cell cycle protein cyclin D1/E1 were determined by WB(F) and qPCR(G) in LN18, T98G, and HS683 with indicated modifications. Statistical results are presented as mean ± SD, *p < 0.05, **p < 0.01, ***p < 0.001, ****p < 0.0001. Data are representative from at least three experiments with similar results. Error bars represent independent experiments.

To investigate the expression of CARD16 in cell lines, WB and qPCR analysis were conducted. The results revealed a significant upregulation of CARD16 expression in GBM cells and GSCs, particularly in LN18 and T98G, when compared to normal human astrocytes (NHA). In contrast, no significant change was observed in the oligodendroglioma cell line HS683 (Fig. 2A, B). Subsequently, stable knockdown models of CARD16 were generated in LN18 and T98G cells (sh1, sh2), along with their respective normal control (NC). Additionally, stable over-expression vector (OV) model of CARD16 were established in HS683 cells, alongside their empty-control vector (EV) (Fig. 2C, D). Considering the structural similarities among CARD-containing proteins, the specificity of CARD16-shRNAs was verified. The potential knockdown effects of CARD16-shRNAs on other CARD-containing genes were excluded via qPCR and WB assays (Supplementary Fig. S2B, C).

To shed light on the inherent biological mechanisms, the CGGA was interrogated for co-expressed genes of CARD16. CARD16 is known to recruit RIPK2 via CARD-CARD domains, ultimately activating NF-kB [22]. Activation of NF-kB is crucial as it promotes the maintenance of stemness and induces resistance to temozolomide in glioma [22–24]. In addition, VEGFA is considered the primary mediator in tumor angiogenesis induced by hypoxia [25], and one of the downstream genes of NF-kB, leading to neovascularization and apoptotic resistance in GBM [26]. The gene co-expression analysis unveiled a positive correlation between the expression levels of CARD16 and NF-κB1 (R = 0.58, *p* < 0.0001) as well as VEGFA (R = 0.55, *p* < 0.0001), respectively (Fig. 2E). The statistical data obtained from qPCR demonstrated a similar trend, reaching significant levels (Fig. 2G). Previous research suggests that inhibition of cyclin D1 expression induces of G0/G1 cell cycle arrest in GBM cells [27]. Moreover, high cyclin E expression is associated with poor prognosis in glioma [28]. Next, for WB assay, the expression levels of NF-κB p65, VEGFA, cyclin D1, and cyclin E1 were significantly enhanced in the NC group compared to the sh groups in LN18 and T98G. Moreover, the expression of these proteins was upregulated in CARD16 OV cells (Fig. 2F). These findings provide evidence suggesting that CARD16 may be implicated with tumorigenesis of human glioma cells.

### CARD16 enhances proliferation, invasion, and migration in human glioma cells

**Fig. 3:**
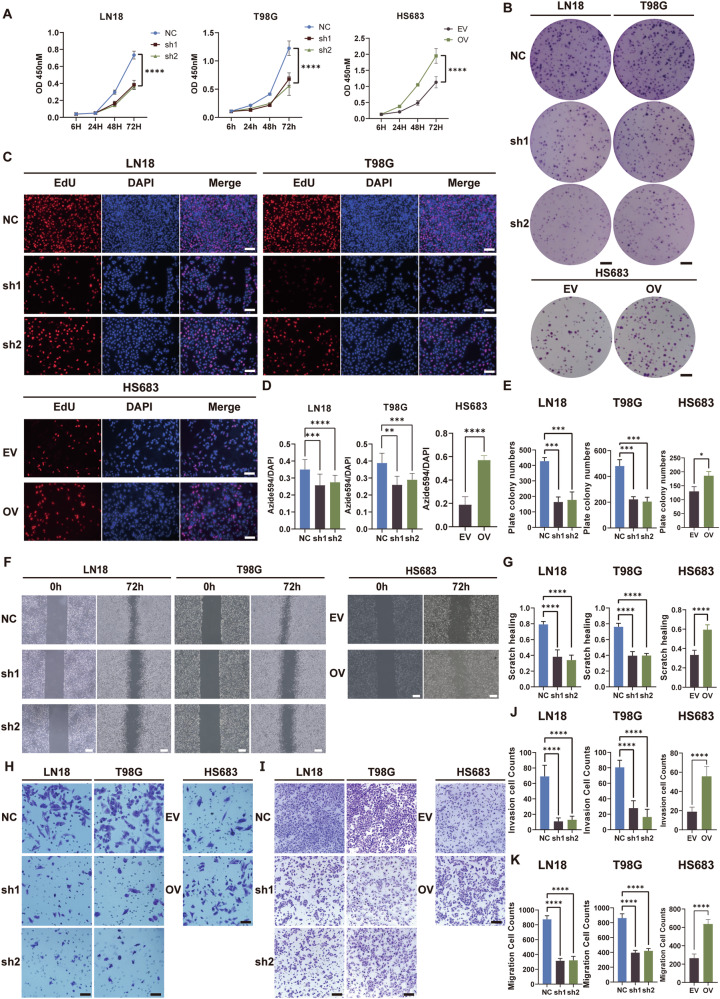
CARD16 enhances proliferation, invasion, and migration in human glioma cells. **A** CCK-8 assay of LN18 and T98G with CARD16 NC and sh cells, and HS683 with CARD16 OV and EV cells. **B** Plate colony formation for testing cell proliferation. Upper, CARD16 NC and sh LN18 and T98G cells were decided. Lower, CARD16 OV and EV HS683 cells were decided. Scale bar, 5 mm. **C** EdU assay for determining cell proliferation. Upper, CARD16 NC and sh LN18 and T98G cells were decided. Lower, CARD16 OV and EV HS683 cells were decided. At least six independent fields of cells were counted and measured. Scale bar, 100 μm. **F** Scratch healing assay to evaluate the cell migration. Left, CARD16 NC and sh LN18 and T98G cells were tested. Right, CARD16 OV and EV HS683 cells were tested. Scale bar, 500 μm. **H** Transwell assay for evaluating cell invasion. Left, CARD16 NC and sh LN18 and T98G cells were decided. Right, CARD16 OV and EV HS683 cells were decided. **I** Transwell assay for evaluating cell migration. Left, CARD16 NC and sh LN18 and T98G cells were decided. Right, CARD16 OV and EV HS683 cells were decided. At least six highly magnifying fields of microscopes were counted and measured in Fig. **H** and **I**. Scale bar, 100 μm. Fig. **D, E, G, J, K** Plate colony numbers, EdU+ cell percentage, scratch healing percentage, invasion cell counts and migration cell counts were analyzed, respectively. Statistical results are presented as mean ± SD, *p < 0.05, **p < 0.01, ***p < 0.001, ****p < 0.0001. Data are representative from at least three experiments with similar results. Error bars represent independent experiments.

To evaluate the impact of CARD16 in cellular proliferation, invasion and migration, a set of experiments were conducted. The findings from CCK-8 and EdU assays indicated that the suppression of CARD16 resulted in a noteworthy reduction in OD450 values and the proportion of EdU-positive cells in GBM. Conversely, the overexpression of CARD16 in HS683 augmented these proliferation-related indicators (Fig. 3A, C, D). In addition, these findings were further confirmed by plate colony formation assays (Fig. 3B, E). The cell cloning efficiency was lower in the sh groups in LN18 and T98G, whereas an improvement was observed in the OV group in HS683.

Subsequently, scratch healing presented a lower percentage of healing in CARD16-sh cells compared to NC cells. Conversely, the OV group showed a significantly higher healing rate of healing in comparison to EV cells (Fig. 3F, G). Additionally, the outcomes obtained from the transwell invasion assays revealed a reduction in the number of invading cells through the matrigel-covered membrane in CARD16-silenced cells compared to the control groups, and CARD16 overexpression increased cell invasion(Fig. 3H, J). These trends were further corroborated by the results of the transwell migration assay (Fig. 3I, K). In this study, it was observed that depletion of CARD16 had a significant effect in reversing migration characteristics and reducing invasiveness in glioma cells. The results of WB revealed that knockdown of CARD16 inhibited mesenchymal markers, such as N-cadherin, and MMP-2, and increased the expression of epithelial factor E-cadherin (Supplementary Fig. S2A).

In summary, these findings provide evidence that CARD16 promotes proliferation, migration and invasion in human glioma.

### Knockdown of the CARD16 gene induces apoptosis in GBM cells

**Fig. 4:**
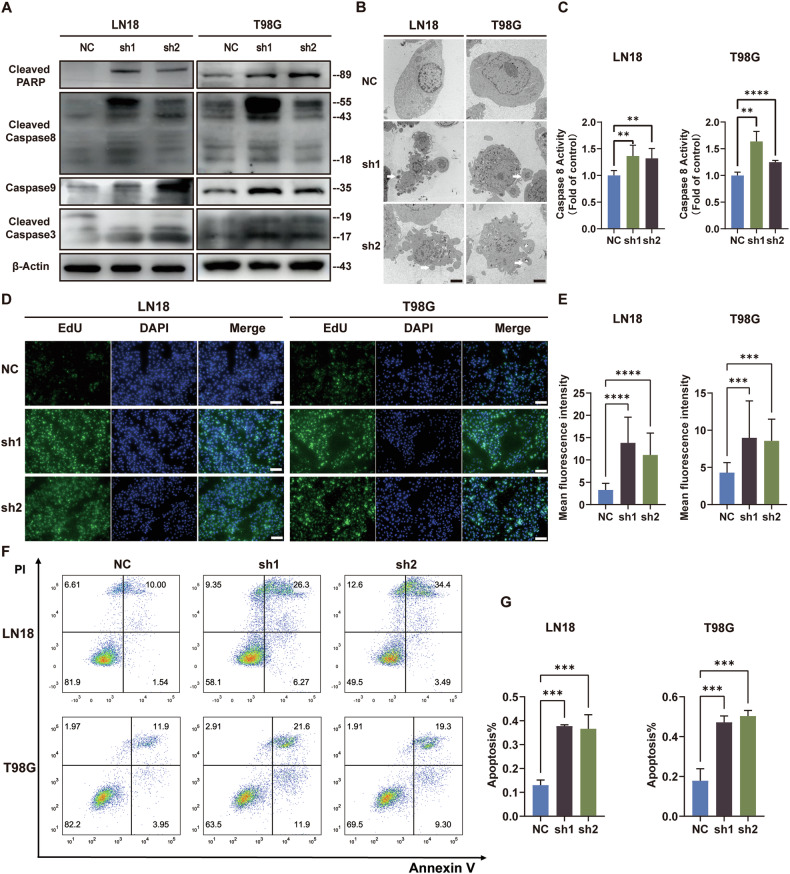
Knockdown of CARD16 enhances GBM cell apoptosis. **A** WB of apoptosis related protein Cleaved PARP, Cleaved Caspase8, Cleaved Caspase9, Cleaved Caspase3 in CARD16 NC and sh LN18 and T98G cells. **B** Typical morphological changes of apoptosis such as apoptotic body were found in CARD16 sh LN18 and T98G cells under electron microscopes. Scale bar, 2μm. **C** Caspase8 activity were measured in CARD16 NC and sh LN18 and T98G cells. **D**, **E** Tunel assay of CARD16 shRNA transfected LN18 and T98G cells and their control cells were decided. At least six independent fields of cells were measured. Scale bar, 100 μm. **F, G** Cell apoptosis were measured by cell cytometry of LN18 and T98G with CARD16 knockdown and their normal control. Statistical results are presented as mean ± SD, *p < 0.05, **p < 0.01, ***p < 0.001, ****p < 0.0001. Data are representative from at least three experiments with similar results. Error bars represent independent experiments.

One of the main functions of CARD is to regulate caspase activation [29]. Specifically, CARD-only proteins (COPs) have been demonstrated to interact with apoptosis-associated speck-like protein containing a CARD (ASC) through homotypic CARD-CARD interactions, thereby regulating over inflammation and apoptosis [20]. To investigate the activation of caspase, the expression of cleaved caspase and the activity of caspase-8 were examined (Fig. 4A, C). In addition, typical apoptotic bodies were observed in CARD16 knockdown cells (Fig. 4B).

To further determine the anti-apoptotic function of CARD16, TUNEL assays were conducted on cell slides. After inducing apoptosis, the percentage of TUNEL positive cell and mean fluorescence intensity in the CARD16-sh GBM cells were increased, significantly (Fig. 4D, E). Furthermore, flow cytometry analysis with Annexin V-PI double staining confirmed that silencing CARD16 significantly induced cell apoptosis in GBM cells (Fig. 4F, G). These findings suggest that knockdown of CARD16 accelerates cell apoptosis in GBM cells.

### FOXO1 is a downstream target of CARD16 in GBM cells

**Fig. 5:**
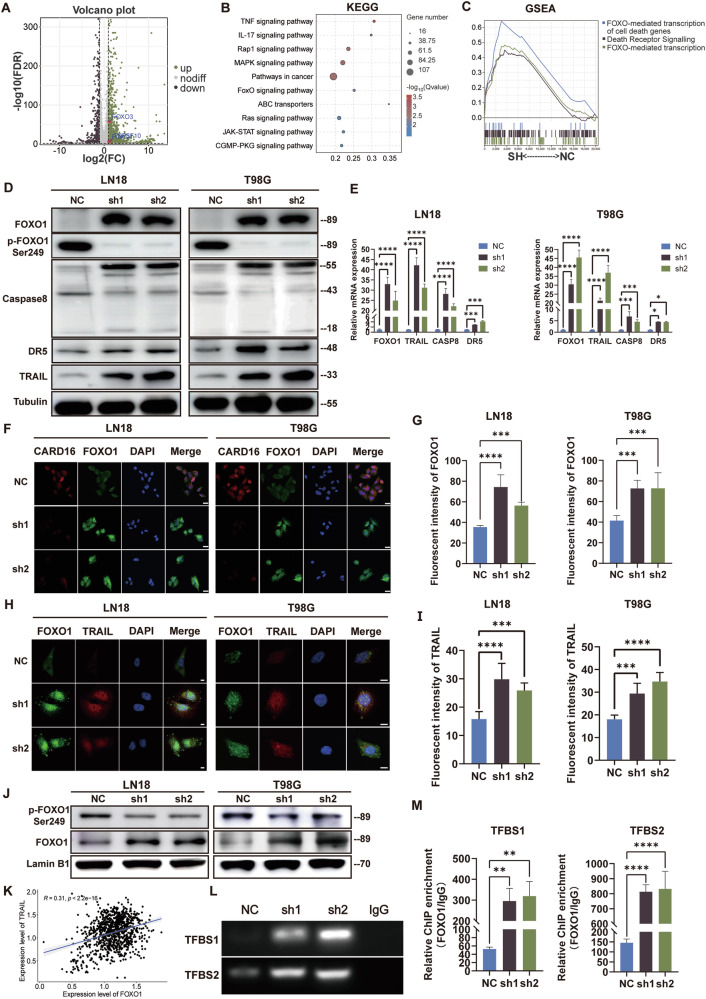
FOXO1 is downstream targets of CARD16 in GBM cells. **A** Volcano plot of DEGs in CARD16 knockdown LN18 and control cells. FOXO1, FOXO3 and TNFSF10(TRAIL) were upregulated in CARD16 knockdown LN18. **B** KEGG enrichment in CARD16 knockdown LN18 was performed. TNF signaling and FOXO signaling were enriched. **C** CARD16-related enrichment plots were shown in GSEA enrichment. Compared to LN18 NC, FOXO-related transcription and programmed cell death were upregulated in CARD16 knockdown LN18. **D, E** WB and qPCR of FOXO1, Caspase8, DR5, and TRAIL in LN18 and T98G with CARD16 knockdown and their normal control. **F, G** Immunofluorescence was performed using anti-CARD16 and anti-FOXO1 antibody in CARD16 shRNA transfected LN18 and T98G cells and their control cells. Scale bar, 20 μm. **H, I** Immunofluorescence was performed using anti-FOXO1 and anti-TRAIL antibody in CARD16 shRNA transfected LN18 and T98G cells and their control cells. At least six highly magnifying fields of microscopes were counted and measured in Fig. F and H. Scale bar, 10 μm. **J** Localization of FOXO1 and p-FOXO1 in the nucleus was decided by WB assay using nuclear protein. **K** Correlation of FOXO1 and TNFSF10 expression in the CGGA databases. **L** Abundance of transcription factor binding site (TFBS) 1 and 2 after PCR were indicated through Agarose gel electrophoresis. **M** Anti-FOXO1/IgG ChIP enrichment of TFBS 1 and 2 were confirmed by ChIP-qPCR.Statistical results are presented as mean ± SD, *p < 0.05, **p < 0.01, ***p < 0.001, ****p < 0.0001. Data are representative from at least three experiments with similar results. Error bars represent independent experiments.

To investigate the downstream targets influenced by CARD16, RNA-sequencing were performed. Figure 5A showed the DEGs. Subsequently, the DEGs were subjected to KEGG, GSEA, and DO analyses. KEGG enrichment revealed that CARD16 was associated with tumorigenesis and apoptosis related pathways, including TNF signaling pathway and FOXO signaling pathway (Fig. 5B). In addition, GSEA demonstrated upregulation of FOXO-mediated Transcription and programmed Cell Death in CARD16 knockdown LN18 cells (Fig. 5C and Supplementary Fig. S3A). DO analysis showed that CARD16 was closely related to central nervous system tumor and malignant glioma (Supplementary Fig. S3B). As two pivotal molecules of the FOXO pathway, FOXO1 and TRAIL were significantly upregulated in the CARD16-sh cells. Furthermore, the expression level FOXO1 was downregulated in glioma (Supplementary Fig. S3C, D, E).

To examine the potential regulatory role of CARD16 in GBM, the activation of the FOXO signaling pathway was detected. The results revealed significant enhancement of FOXO1, Caspase 8, DR5, and TRAIL, while downregulation of p-FOXO1 Ser249 in GBM cells with CARD16 knockdown (Fig. 5D, E). In addition, fluorescence images demonstrated that FOXO1 was markedly upregulated with CARD16 downregulation (Fig. 5F, G). Meanwhile, the co-expression of FOXO1 and TRAIL was observed in CARD16 knockdown cells (Fig. 5H, I). Interestingly, a concentrated signal of FOXO1 in the nucleus was noticed (Fig. 5H). Consequently, the nucleoprotein was isolated from the cell lysate. Western blotting demonstrated an increased localization of FOXO1 in the nucleus of CARD16 downregulated cells (Fig. 5J). To validate the above findings, the CGGA database was reexamined and the coexpression of FOXO1 and TRAIL was identified (Fig. 5J). Subsequently, the JASPAR database was interrogated for the binding sequence of FOXO1 and the TFBSs on the promoter of TRAIL (Supplementary Fig. S3F, S3G). Binding efficency of FOXO1 on TFBS1 and TFBS2 was verified by ChIP-qPCR. Following this, X-ChIP was performed in the LN18-NC and sh cells. The results of ChIP-qPCR and AGE revealed an upregulation of FOXO1-TFBS binding in the TRAIL promoter in CARD16-sh cells (Fig. 5L, M).

Collectively, these data imply that knockdown of CARD16 leads to upregulation of FOXO1 expression and facilitates the translocation of FOXO1 into the nucleus for transcriptional regulation.

### Depletion of FOXO1 restores proliferation and reduces apoptosis of GBM

**Fig. 6:**
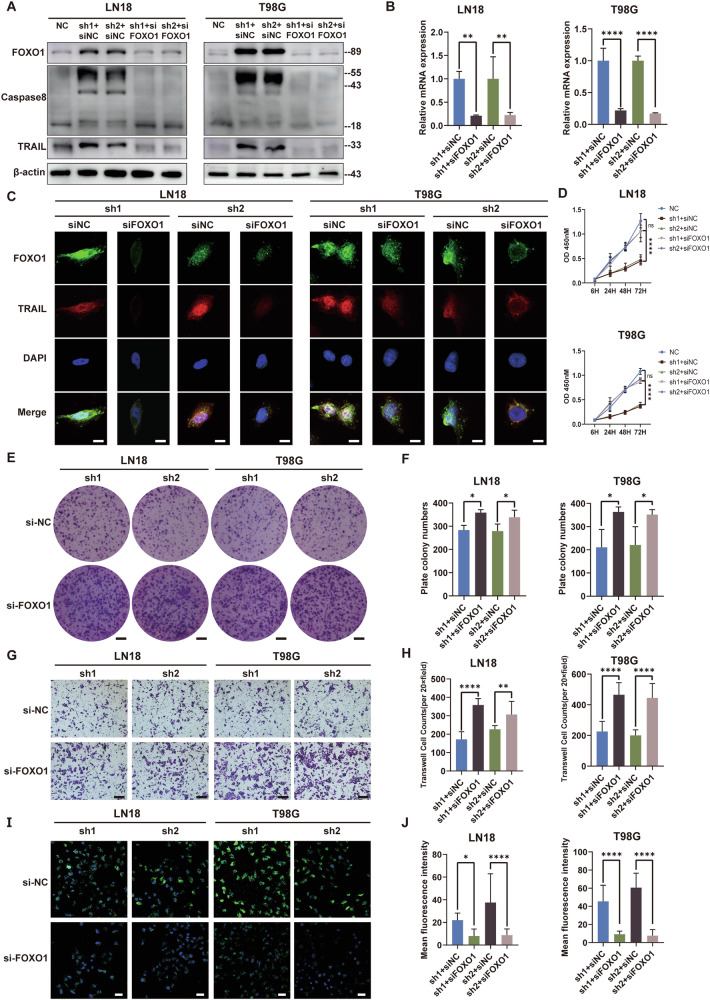
Depleting FOXO1 restores proliferation and reduces apoptosis of GBM. **A** WB were preformed to detect the expression of FOXO1, Caspase 8, and TRAIL in siFOXO1 transferred CARD16 knockdown LN18 and T98G. **B** FOXO1 mRNA expression level was decided through qPCR in siFOXO1 transferred CARD16 knockdown LN18 and T98G. **C** Immunofluorescence was performed using anti-FOXO1 and anti-TRAIL antibody in siFOXO1 transferred CARD16 knockdown LN18 and T98G and their control cells. Scale bar, 10 μm. **D** Cell proliferation of CARD16 sh cells and NC. **E**. Plate colony formation of siFOXO1 transferred CARD16 sh and NC cells. Scale bar, 5 mm. **G** Cell migration assay of siFOXO1 transferred CARD16 sh and NC cells. Scale bar, 100 μm. **I** Tunel assay of siFOXO1 transferred CARD16 sh and NC cells. Scale bar, 50 μm. Fig. **F**, **H**, **J**. Plate colony numbers, migration cell counts, and mean fluorescence intensity of Tunel assay were analyzed. Statistical results are presented as mean ± SD, *p < 0.05, **p < 0.01, ***p < 0.001, ****p < 0.0001. At least six highly magnifying fields of microscopes were counted and measured. Data are representative from at least three experiments with similar results. Error bars represent independent experiments.

To gain deeper insights into the functional alterations in GBM cells induced by CARD16 via the FOXO signaling pathway, we introduced FOXO1 siRNA(siFOXO1) and a normal control (siNC) into CARD16 knockdown LN18 and T98G cells, respectively (Fig. 6B). Subsequently, we examined the expression levels of FOXO1, TRAIL, and Caspase 8, and found that the silencing of FOXO1 counteracted the upregulation of these proteins (Fig. 6A). This finding was further supported by IF staining for FOXO1 and TRAIL (Fig. 6C). Additionally, cellular functions of proliferation and migration were assessed using the CCK-8 assay (Fig. 6D), plate colony assay (Fig. 6E, F), and Transwell migration assay (Fig. 6G, H). It was observed that the decrease in cellular functions caused by CARD16 knockdown was restored after silencing FOXO1. Additionally, to evaluate the anti-apoptotic cellular ability, we performed a TUNEL assay, which revealed that the silencing of FOXO1 reduced the percentage of apoptotic cells under CARD16 knockdown conditions (Fig. 6I, J). Furthermore, FACS analysis with AnnexinV-PI yielded similar results (Supplementary Fig. S4A, B).

Taken together, the aforementioned results demonstrate that the knockdown of CARD16 inhibits the tumorigenic effects mediated by upregulating FOXO1 in glioma cells. The tumor apoptotic effects exerted by TRAIL are also dependent on the FOXO1.

### Downregulation of CARD16 restricts the tumorigenicity of GBM cells in vivo

**Fig. 7:**
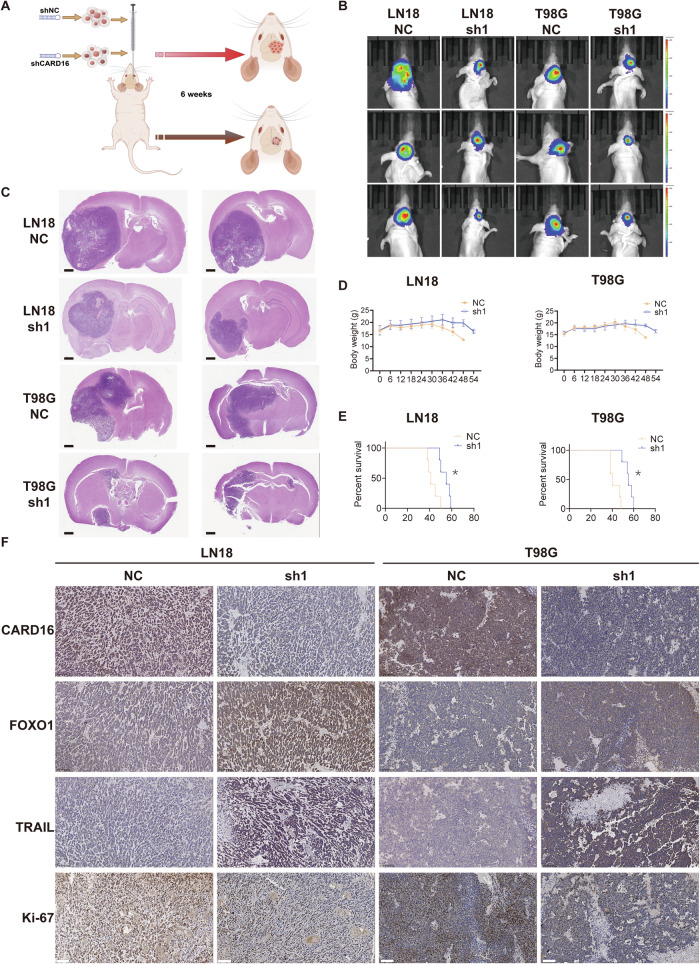
Downregulation of CARD16 restricts the tumorigenicity of GBM cells in vivo. **A** Intracranial tumor formation patterns in nude mice. **B** Representative images of in vivo fluorescence assay on days 42 post-implantation using LN18 and T98G with indicated modifications. Each group contains 5 mice. **C** Representative images of HE staining of mouse brains harvested on days 35 after in vivo fluorescence assay. Scale bar, 1 mm. **D** Body weight change curves of nude mice with intracranial tumors. Each group contains 5 mice. **E** Survival curves calculated using the Kaplan-Meier method of nude mice with intracranial tumors. Each group contains 5 mice. **F** Representative immunohistochemistry (IHC) images of CARD16, FOXO1, TRAIL, and Ki-67 expression in the above mice. Scale bar, 100 μm. Statistical results are presented as mean ± SD, *p < 0.05,. Data are representative from at least three experiments with similar results.

The effect of CARD16 in a mouse xenograft glioma model was investigated (Fig. 7A). GBM cells with sh1, along with their respective control were selected for intracranial xenograft due to their relatively higher efficiency. The examination of tumor sizes utilizing bioluminescence imaging showed a reduction in animals bearing sh1 cells (Fig. 7B), and tumors in the sh1 group appeared smaller based on H&E staining (Fig. 7C). Additionally, the sh1 group exhibited slower weight loss and longer survival time compared to the control group (Fig. 7D, E). Subsequently, mouse brain tissues were subjected to IHC staining. Compared to the NC groups, CARD16 staining was notably diminished, whereas FOXO1 and TRAIL staining were significantly enhanced, compared to the NC groups. IHC staining also confirmed the low expression level of Ki-67 in the sh group (Fig. 7F). Overall, these results demonstrate that knocking down CARD16 inhibits the tumorigenicity of GBM cells in an in vivo setting.

### CARD16 knockdown diminishes CDK2-mediated ubiquitination of FOXO1

**Fig. 8:**
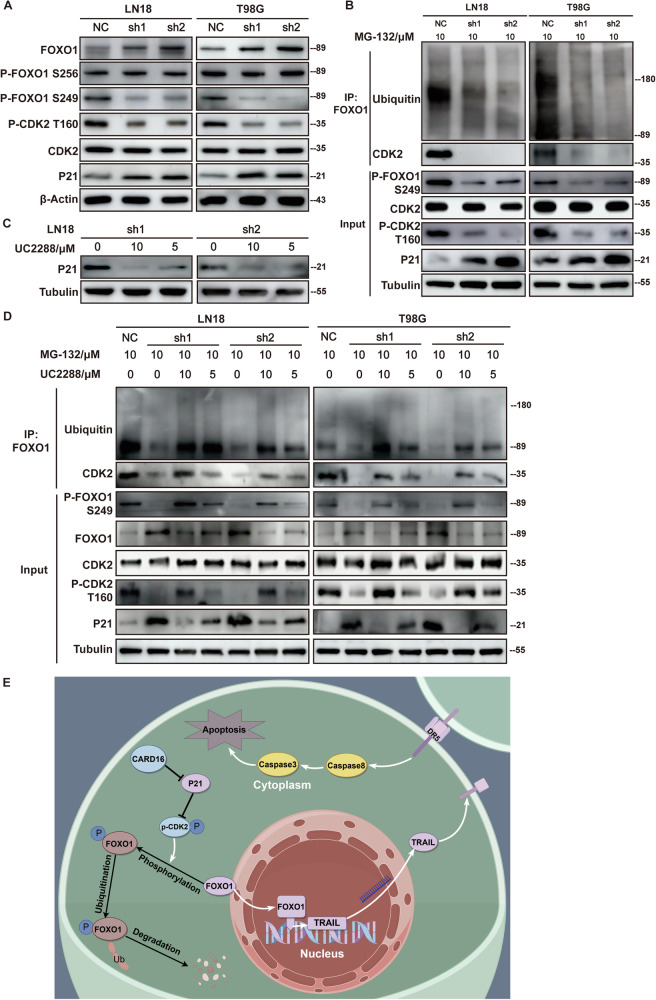
CARD16 knockdown diminishes CDK2-mediated ubiquitination of FOXO1. **A** WB were preformed to detect the expression of FOXO1, p-FOXO1 Ser256, p-FOXO1 Ser249, p-CDK2 Thr160, CDK2, and P21 in CARD16 knockdown LN18 and T98G. **B** Equal quantities of cells were cultured in complete medium supplemented with MG132 for a duration of 8 h. Subsequently, the cells were lysed for IP and WB with the indicated antibodies. **C** Cells were cultured in complete medium supplemented with UC2288 for 16 h, followed by MG132 treatment for 8 h and lysed for IP and WB. **D**. IP and WB assay using lysate from NC and sh cells supplemented with UC2288 and MG132. Data are representative from at least three experiments with similar results. **E** The schematic diagram shows the proposed mechanism that CARD16 restores tumorigenesis and restraints apoptosis through FOXO/TRAIL axis in glioma.

Previous studies have provided evidence that the phosphorylation of FOXO1 is facilitated by the activation of protein kinase B (AKT) signaling, primarily targeting the Ser256 residue, and cyclin-dependent kinases 2 (CDK2), which predominantly acts on the Ser249 residue [30, 31]. This phosphorylation process ultimately results in the degradation of FOXO1 via ubiquitination [32]. The evaluation conducted on the Akt and CDK signaling indicated that Akt and p-FOXO1 Ser256 was not inhibited in the CARD16-sh GBM cells (Supplementary Fig. S2B, S2D, S5A). Conversely, there was a substantial enhancement observed in the expression level of CDK Inhibitor 1 A (CDKN1A/P21) [33] in the CARD16-sh GBM cells(Supplementary Fig. S5A), and phosphorylation of endogenous FOXO1 at Ser249 was abolished (Fig. 8A). To validate the interaction modification of FOXO1 and CDK2 in GBM cells, endogenous FOXO1 proteins were subjected to IP assay with FOXO1 antibody and immunoblotted with ubiquitin and CDK2 antibodies. The results indicated that in CARD16-sh GBM cells the CDK2-FOXO1 interaction and ubiquitination of the endogenous FOXO1 protein were suppressed (Fig. 8B). To further confirm the role of P21 in reducing endogenous FOXO1 ubiquitination, we employed the small-molecule inhibitor UC2288 to suppress P21 expression and activity [34] (Fig. 8C), and subjected these cells to IP. The results showed that UC2288 significantly enhanced the interaction between CDK2 and FOXO1, leading to an augment of phosphorylation of FOXO1 in Ser249 residue. Consequently, there was an observed elevation in the levels of FOXO1 ubiquitination, accompanied by a corresponding decline in FOXO1 protein levels (Fig. 8D).

These findings collectively imply that the downregulation of CARD16 inhibits the phosphorylation and ubiquitination of FOXO1, possibly as a result of decreased P21 levels and enhanced CDK2 activity.

## Discussion

Abnormal genetic alterations in glioma lead to uncontrolled, unrestricted, and accelerated cell proliferation through dysregulated mechanisms of cellular proliferation and apoptosis [35]. ASC has been identified as a tumor suppressor, and a promoter of apoptotic pathways [36, 37]. Latest research has demonstrated that CARD16 exerts the strongest regulation on ASC polymerization among the COPs [20], implying CARD16 acting as an apoptosis regulator. However, the role of CARD16 in cancers is less well described. Focusing on the DEGs and survival-related genes in glioma, our findings revealed that CARD16 expression is enhanced in malignant glioma, particularly in GBM. Previous study reported that CARD16 activates NF-κB via interacting CARD-containing proteins [22]. NF-κB activation is crucial in gliomagenesis and inhibition of apoptosis [24, 38], in addition to angiogenesis through VEGF pathway [39]. Our findings confirmed that downregulating of CARD16 attenuates the expression of NF-κB p65 and VEGFA in GBM cells. Likewise, the cell function experiments provide findings that CARD16 plays an important role in maintaining proliferation, promoting migration, and mitigating apoptosis in glioma. Additionally, the expression levels of cell cycle (Cyclin D1/E1), metastasis (E-Cadherin/N-Cadherin), and apoptosis (cleaved Caspase-3,8,9) related proteins enhance the strength of the evidence. Moreover, the xenograft model corroborated these experimental findings. Based on our results, we postulate that CARD16 facilitates tumorigenesis and impedes apoptosis in glioma. Nevertheless, further investigation is warranted to elucidate the underlying molecular mechanism.

Consequently, gene expression changes were detected in LN18 cells (NC and sh1). The KEGG enrichment analysis revealed significant alterations in signaling pathways, specifically the TNF and FOXO signaling pathways, which are well-established in their associations with tumorigenesis. Furthermore, GSEA indicated enrichment of FOXO-mediated Transcription and Death Receptor signaling. Previous studies have reported a decrease in FOXO1 expression in glioma, and our research confirms this trend using online databases and clinical specimens. Post-transcriptional modifications of FOXO1, such as acetylation [40], phosphorylation [41], and noncoding RNA [42, 43], play a substantial role in gliomagenesis. The phosphorylation of FOXO1 results in the degradation via ubiquitination [32]. Interestingly, upregulation of FOXO1 can significantly reduce the tumorigenicity of GBM and improve survival [17, 40]. TRAIL, an apoptosis inducer specific to tumors without affecting normal cells, h has been explored for glioma management [9, 10, 44]. However, the high resistance of TRAIL signaling in GBM and the presence of the blood-brain-barrier pose significant obstacles to the clinical application of TRAIL-based GBM therapy [44, 45]. The transcription of TRAIL can be ameliorated by the binding of FOXO1 to the TRAIL promoter. Accordingly, the expression of TRAIL is amplified, thereby activating the extrinsic apoptotic pathway [46, 47]. In recent research, we reported a significant upregulation of the FOXO1/TRAIL axis in GBM cells following CARD16 downregulation. Furthermore, we confirmed the augmentation of FOXO1 in the nucleus, which promotes TRAIL transcription. Additionally, silencing of FOXO1 effectively reversed cell cycle arrest and apoptosis, suggesting that activation of the FOXO1/TRAIL axis plays a crucial role in regulating of cellular fate in GBM.

The main limitation of our study is that the direct interaction between CARD16 and FOXO1 has not been discovered. Previous literatures have indicated that the activation of the Akt pathway leads to the phosphorylation of FOXO1 at the Ser256 residue [30] while CDK2 targets the Ser249 residue [31]. In the present study, it was confirmed that Akt and phosphorylation of FOXO1 at Ser256 did not exhibit a decrease in the CARD16 knockdown GBM cells. However, the CDK2 inhibitor, P21 exhibits a significantly higher expression level in the CARD16-sh cells. P21 has been established as a p53-independent tumor suppressor and an apoptosis-promoting molecule in GBM [48]. Meanwhile, the phosphorylation of FOXO1 at Ser249 exhibits significant restraint, while the ubiquitination of FOXO1 shows attenuation in the CARD16-sh cells. These trends can be restored by P21 inhibitor UC2288. The regulatory mechanism of P21 in cancer is a subject of complexity [33]. In this study, we have provided evidence illustrating that the knockdown of CARD16 results in the up-regulation of P21 in GBM. Additionally, it activates FOXO1 and the subsequent pathways by inhibiting phosphorylation of FOXO1 mediated by CDK2. Nevertheless, the direct mechanism through which CARD16 regulates P21 remains to be fully elucidated in our next investigation.

According to above evidence, CARD16 is increased in malignant glioma and functions as a tumor promoter in glioma through enhancing phosphorylation and ubiquitination of FOXO1, and additionally limiting TRAIL-induced apoptosis. CARD16 has huge prospects in prognosis prediction and gene therapeutics.

## Materials and methods

### In-silico analysis

DEGs were obtained: GBM samples (*n* = 697) from The Cancer Genome Atlas (TCGA) database, normal brain tissues (*n* = 1153) from the Genotype-Tissue Expression Project database (GTEx), and glioma samples (*n* = 1018) from the Chinese Glioma Genome Atlas (CGGA) database. The survival data were obtained from the CGGA and TCGA databases. The collected data was then analyzed using R studio. The co-expression between two genes was assessed using Pearson statistics.

### Clinical specimens

The study was approved by the Ethics committee of the First Affiliated Hospital of Sun Yat-sen University (FAH of SYSU) and conducted in accordance of the Declaration of Helsinki (No. 2020322). Fresh specimens and glioma pathological section were obtained from inpatients at the Department of Neurosurgery at the FAH of SYSU (Supplementary Table S1). Informed consent was obtained from all patients. The characterizations of the tumor and peritumoral tissues (peritumoral edema zone) were accomplished through the utilization of both rapid frozen pathology and routine paraffin pathology techniques.

### Immunohistochemistry (IHC) and H&E staining

Paraffin embedded sections were heated, deparaffinized, rehydrated using sequential concentrations of xylene and ethanol. After antigen repair using sodium citrate buffer, the slides were incubated with primary antibodies at 4 °C overnight. The signal was visualized using secondary antibodies and 3,3′-diaminobenzidine (DAB) as the substrate. Subsequently, the slides were counterstained with hematoxylin. Sections were utilized immediately for hematoxylin and eosin staining subsequent to gradient hydration. Images were obtained using a Kfbio/KF-PRO-020 scanner.

### Western blotting (WB)

Proteins were extracted and quantified by BCA method (Beyotime, Shanghai, China). 7–12% SDS-PAGE gels were used to load denatured protein. After electrophoresis, membrane transfer and blocking, membranes were incubated with the primary antibodies overnight at 4 °C. The secondary antibodies (Invitrogen, Carlsbad, CA, USA) were incubated at room temperature (RT) for one hour. The signals were observed by enhanced chemiluminescence using ECL Substrate (Bio-Rad, Shanghai, China).

### Cell cultures and treatments

293 T cell was purchased from ATCC. Normal human astrocytes (NHA) and human glioma cell lines HS683, U251, U118, LN18, T98G were kindly provided by Dr. Suyun Huang, Virginia Commonwealth University. These Cells were cultured in Dulbecco’s modified-Eagle’s medium (DMEM) with 10% fetal bovine serum(FBS). Glioma stem cells (GSCs) MES28 and GSC23 were kindly provided by Dr. Jeremy N. Rich. These cells were cultured in DMEM/F12 medium with B27, EGF and β-FGF (20 ng/ml each). Cells were cultured at 37 °C in a humidified atmosphere of 5% CO_2_ and 95% air.

### Immunofluorescence (IF) staining

Cells on coverslips were fixed with 4% paraformaldehyde and permeabilized using 0.3% Triton. Incubation with primary antibodies was performed overnight at 4 °C, followed by incubation with fluorescent secondary antibodies. Nuclei were counterstained with 4’,6-diamidino-2-phenylindole (DAPI). Images were acquired using a Airyscan confocal microscope.

### Antibodies

The primary antibodies are depicted in Supplementary Table Materials.

### Quantitative polymerase chain reaction (qPCR)

Reverse transcription was carried out with Evo-M-MLV reagent (Accurate-Biology, Hunan, China). SYBR-Green Premix was used in qPCR. The primer sequences and parameters are listed in Supplementary Table Materials. Fold changes were measured after qPCR.

### Small interference RNA (siRNA) transfection

SiRNAs targeting CARD16 and FOXO1 were synthesized by GenePharma (Suzhou, China). The siRNAs were transfected with Lipofectamine 3000 reagent. The siRNA sequences are listed in Supplementary Table Materials.

### Lentivirus production and stable cell line establishment

Lentivirus with CARD16-shRNA vectors and CARD16-overexpression vectors were obtained from GenePharma. The shRNA sequences are listed in Supplementary Table Materials. To establish stable cell lines, glioma cells were transduced with lentivirus in culture medium containing 8 μg/ml polybrene. After 24 h of incubation, cells were screened with 2 μg/ml puromycin for 72 h. The specificity of the shRNA sequences was validated through qPCR using CARD17, CARD18, Caspase-1(CASP1), and ASC/PYCARD primers (Supplementary Fig. S2C).

### Cell counting kit-8 (CCK8) assay

The cells were seeded 3 × 10^3/well in 96-well plates. The Cell Counting Kit-8 (Dojindo, Shanghai, China) was added and the absorbance at 450 nm wavelength (OD450) was measured at 6, 24, 48, 72 h after paving.

### 5-ethynyl-2′-deoxyuridine (EdU) assay

After incubated with EdU for 2 h, cells were fixed for 20 min, permeabilized for 10 min, and incubated with Click reaction mix (Beyotime) for 30 min. The nuclei were stained with DAPI. Representative images were obtained using a fluorescence microscope.

### Colony formation assay

1 × 10^3 cells were cultured in six-well plates in medium with 10% FBS for 2 weeks. Cells were fixed with 4% paraformaldehyde for 15 min and stained with 0.1% crystal violet for 15 min. Colonies were photographed using a multi-function imager.

### Scratch healing assay

3 × 10^5 Cells were cultured in six-well plates overnight. After scratched the cells were cultured in DMEM for 48 h. The pictures were taken with a microscope at 0, 24, and 48 h after scratch. Scratch area was calculated by Fiji-Imagej software.

### Transwell invasion and migration assays

Complete medium was added to the lower chambers and serum-free medium with 10^4 cells was added to the 8.0μm pore chambers (Corning, NY, USA). After 24 h, migratory cells were fixed by 4% paraformaldehyde and stained using 0.1% crystal violet. The invasion assay was performed following the methodology, except adding 50 μl Matrigel (Corning) to upper chamber.

### Flow cytometry (FACS)

After digestion and centrifugation, cells were resuspended in 195 µL binding buffer (Beyotime) with 5 µL AnnexinV-FITC and 10 µL PI. Followed by avoidance reaction for 10 min, FACS was carried out using LSRFortessa (BD).

### TdT-mediated dUTP Nick-End Labeling (TUNEL) apoptosis assay

Briefly, the cells were pre-treated with 0.3% H_2_O_2_ for 2 min. The TUNEL assay was conducted using the TUNEL Apoptosis Detection Kit (Vazyme, Nanjing, China) according to the manufacturer’s protocol.

### Caspase 8 activity assay

Caspase activity was decided using Caspase-8 Assay Kit (Beyotime) according to the manufacturer’s instructions. The release of p-nitroanilide was qualified by the absorbance at 405 nm.

### Transmission electron microscopy

Cells were harvested and fixed in a paraformaldehyde-glutaraldehyde buffer (Servicebio, Wuhan, China). The cells were then suspended in 1% agarose, and fixed with 1% osmic acid for 2 h. The cell sediment was gradient dehydrated, infiltrated and embedded in the SPI-PON-812(SPI, WC, PA, USA). After lead citrate staining, cells were observed under HT7700 transmission electron microscopy.

### RNA sequencing (RNA-seq)

The total mRNA was extracted from LN18-NC and LN18-sh1 cells. High throughput RNA-seq was performed by Illumina Nova 6000 (Genedenovo, Guangzhou, China). *P*-value < 0.05 and fold change cutoff of log2 ratio >1 was employed to identify DEGs (Supplementary Table S2). The analysis of DEGs was conducted using the Omicsmart platform (www.omicsmart.com).

### Agarose gel electrophoresis (AGE)

DNA marker and DNA samples were diffused in 1.5% agarose gel under 100–120 V electrophoresis. A UVP GelStudio was used to visualize DNA bands.

### Chromatin immunoprecipitation (ChIP)

Cells were cross-linked in 1% formaldehyde and blocked by glycine. Chromatin was extracted using the Chromatin Extraction Kit (abcam, Cambridge, UK) and sheared to 200–500 bp fragments using Bioruptor Plus (Diagenode, Belgium). ChIP was performed by the ChIP Kit(abcam). JASPAR database(https://jaspar.elixir.no/) was interrogated to predict transcription factor binding sites (TFBSs). The sequences of TFBSs and their primers were listed in Supplementary Table Materials.

### Immunoprecipitation (IP)

Cells were suspended in RIPA Lysis Buffer(Beyotime) for 30 min on ice. Lysates were centrifuged at 12,000 *g* for 10 min, and 1 μL indicated antibodies was added to supernatant. After rotated overnight, incubate 25 μL protein A/G agarose beads (Invitrogen) for 4 h at 4 °C. Afterward, beads were incubated with SDS-loading buffer and boiled for WB assays.

### Inhibitors

MG132 and UC2288 were purchased from MCE and dissolved in dimethyl sulfoxide (DMSO).

### Intracranial xenograft experiments

Animal experiments were approved by the Sun Yat-Sen University’s Institutional Animal Care and Use Committee (No.2021173). Animal care and ethics were as previously described [49]. Four-week-old female BALB/c nude mice were divided randomly into four groups (*n* = 10). 5 × 10^4 glioma cells bearing luciferase were injected into the right frontal lobe. After six-weeks of feeding, mice were selected at random to receive an intraperitoneal injection of VivoGloTM solution (Promega, Madison, WI, USA, 10 µl/g). Imagines were obtained using IVIS Lumina. Brain tissues were harvested and embedded for further experiments. The remaining mice were fed routinely and the dates of death were recorded.

### Statistical analysis

Experimental data were expressed as the mean ± standard deviation (SD) of at least three biological replicates. Differences were analyzed by two-sided Student’s *t* test or two-way ANOVA. The log-rank test using Kaplan-Meier method was performed to compare survival curves calculated. The significance level was set at *P* < 0.05. Statistical tests were conducted using GraphPad Prism9.

## Supplementary information

Supplementary Materials

Supplementary Figure. S1

Supplementary Figure. S2

Supplementary Figure. S3

Supplementary Figure. S4

Supplementary Figure. S5

Supplementary Table S1

Supplementary Table S2

Supplementary Original WB

## Data Availability

The datasets analysed during the current study are available in the Genome Sequence Archive (GSA) repository, BioProject: PRJCA031752 [https://ngdc.cncb.ac.cn/gsa/]. The RNA-seq data analysed during this study are also included in Supplementary Table S2.
