# Supplementary material for: CARD16 restores tumorigenesis and restraints apoptosis in glioma cells Via FOXO1/TRAIL axis
Source: Cell Death Dis. 2024 Nov 8;15(11):804. doi: 10.1038/s41419-024-07196-2 (PMC11549220; doi:10.1038/s41419-024-07196-2)
Supplement: Supplementary file 1 — Supplementary Materials [file 41419_2024_7196_MOESM1_ESM.pdf]

CARD16 Restores Tumorigenesis and Restraints Apoptosis in Glioma Cells Via FOXO1/TRAIL Axis

Supplementary Materials

1. The information and concentrations of primary antibodies

Anti-Bodies	Method	Concentration	Manufacturer	Art.No.
CARD16	WB	1:1000	SAB	46403
CARD16	IHC	1:200	SAB	46403
Ki-67	IHC	1:200	Abcam	ab15580
CARD16/COP1	IF	1:200	Abnova	H00114769-B01P
Caspase 8/p43/p18	WB	1:1000	proteintech	66093-1-Ig
DR5	WB	1:1000	proteintech	15497-1-AP
β-Actin	WB	1:5000	proteintech	66009-1-Ig
FOXO1	WB	1:1000	ImmunoWay	YT1758
FOXO1	IHC/IF	1:200	ImmunoWay	YT1758
FOXO1	ChIP	1:50	ImmunoWay	YT1758
FOXO1	IP	1:200	proteintech	18592-1-AP
TRAIL	WB	1:1000	ImmunoWay	YT5511
TRAIL	IHC/IF	1:200	ImmunoWay	YT5511
Lamin B1	WB	1:1000	ImmunoWay	YT5180
Cleaved Caspase-3	WB	1:1000	Abcam	9661S
Cleaved PARP	WB	1:1000	Abcam	5625P
Cyclin D1	WB	1:1000	Abcam	ab134175
VEGFA	WB	1:1000	Abcam	ab1316
β-Tubulin	WB	1:5000	Boster	M05613-4
Cleaved Caspase-9	WB	1:1000	CST	7237S
Cyclin E1	WB	1:1000	CST	4129S
NF-κB p65	WB	1:1000	CST	8242s
P-FOXO1(Ser256)	WB	1:1000	ImmunoWay	YP0113
P-FOXO1(Ser249)	WB	1:1000	ImmunoWay	YP1671
CDK2	WB	1:1000	HuaBio	ET1602-6
P-CDK2(Thr160)	WB	1:1000	HuaBio	HA722582
CDK2	WB	1:1000	Abiowell	AWA02066
E-cadherin	WB	1:5000	HuaBio	ER63312

N-cadherin	WB	1:5000	Proteintech	22018-1-AP
MMP-2	WB	1:1000	Proteintech	10373-2-AP
p-Akt Thr308	WB	1:1000	CST	13038S
p-Akt Ser473	WB	1:1000	CST	4060P
Pan-Akt	WB	1:1000	Boster	BM4400
ASC	WB	1:1000	HuaBio	ET1611-62
CASP1	WB	1:1000	HuaBio	ET1608-69
Ubiquitin	WB	1:1000	HuaBio	ET1609-21
P21	WB	1:1000	Proteintech	10355-1-AP

SAB: College Park, Maryland, USA; Abnova: Taoyuan, Taiwan, China; Immunoway: Plano, Texas, USA; CST: Boston, Massachusetts, USA; Proteintech: Wuhan, Hubei, China; Boster: Wuhan, Hubei, China; HuaBio: Hangzhou, Zhejiang, China; Abiowell: Changsha, Hunan, China.

2. The sequences of primers used for qPCR

Primer	forward sequences (5'to3')	reverse sequences (5'to3')
CARD16 1	TCCGTTATTCCGAAAGGGGC	TTCCAGGTATCGGACCTGCT
CARD16 2	AGACAAGGGTGCTGAACCAG	TTCCAGGTATCGGACCTGCT
PIK3CA	CCACGACCATCATCAGGTGA	CCTCACGGAGGCATTCTAAA
p65/RELA	CCCACGAGCTTGTAGGAAAG	GGATTCCCAGGTTCTGGAAA
NF-κb 1	AACAGAGAGGATTTCGTTTC	TTTGACCTGAGGGTAAGACT
NF-κb 2	ATGGAGAGTTGCTACAACCC	CTGTTCCACGATCACCAGGT
VEGFA	AGGGCAGAATCATCACGAAG	AGGGTCTCGATTGGATGGCA
GADPH	ACAACTTTGGTATCGTGGAAGG	GCCATCACGCCACAGTTTC
β-actin	TGGAACGGTGAAGGTGACAG	AACAACGCATCTCATATTTG
TNFSF10	TGCGTGCTGATCGTGATCTT	GCTCGTTGGTAAAGTACACG
FADD	CGCCTGGGGAAGAAGACCTG	ATTCTCAGTGACTCCCGCAC
CASP8	CTGGTCTGAAGGCTGGTTGT	GTGACCAACTCAAGGGCTCA
DR4	GTTGGTGGCTGTGCTGATTG	TGCGTTGCTCAGAATCTCGT
DR5	CTCCTGAGATGTGCCGGAAG	GGACTTCCCCACTGTGCTTT
FOXO1	GCCACATTCAACAGGCAGCA	TAACTTCGCGGGGCCATCC
PYCARD	TGGATGCTCTGTACGGGAAG	CCAGGCTGGTGTGAAACTGA
CARD17	AAAGCAGTTTATCCGTTCAG	AAGCAAAGCTCGGGCCTTAT
CARD18	TGGGTGCAGGCACAATAAAT	TTGAGGCAAGTTGAGGGTCT
CASP1	TTTCCGCAAGGTTCGATTTT	GGCATCTGCGCTCTACCATC
Akt1	AGCGACGTGGCTATTGTGAA	GCCATCATTCTTGAGGAGGA
CDK2	CCAGGAGTTACTTCTATGCC	TTCATCCAGGGGAGGTACAA
CDKN1A	TGTCCGTCAGAACCCATGC	AAAGTCGAAGTTCCATCGCT
TFBS1	GGTTTCAAAGACACACGGGG	TCATGCTTTGGCTCTCAGCG
TFBS2	AATAGCCAGTTGTCCAACAC	CAGAACCATGAGAGCTTGGT
TFBS3	ACCAAGCTCTCATGGTTCTG	ACCAAGCTCTCATGGTTCTG

3. The procedures of qPCR

Step	Temperature（℃）	Time（s）
Enzyme activation	95	300
Unwinding	95	10
Annealing/extension	60	22-25

4. The sequences of siRNAs and shRNAs used for gene silencing

RNAi	Positive strand（5'—3'）	Negative strand（3'—5'）
CARD16 shNC	GTTCTCCGAACGTGTCACGT	GCACTGTGCAAGCCTCTT
CARD16 shRNA1	CCTGGAAATTAGCTTAGTACA	TGTACTAAGCTAATTTCCAGG
CARD16 shRNA2	GAGGTGCCATGTTCAGAATAC	GTATTCTGAACATGGCACCTC
FOXO1 siRNA	GGAGGUAUGAGUCAGUAUATT	UAUACUGACUCAUACCUCCTT
NC siRNA	UUCUCCGAACGUGUCACGUTT	ACGUGACACGUUCGGAGAATT

5. The table of predicted Transcription factor binding site (TFBS)

NO.	Name	Score	Relative	Start	End	Strand	Sequence
1	MA1947.2	9.443794	0.875636	1285	1294	-	GAAAGCAGGT
2	MA1953.2	9.413919	0.826862	1791	1803	+	ATAAATAGCCAGT
3	MA1955.1	10.461842	0.832603	1939	1952	-	AAAAACATGACGTA

Supplementary Figure Legends

Supplement Figures:

Fig.S1: A. The overlap genes of DEGs and Survival related genes in GBM samples in TCGA database. Figure B, C, D showed the overall survival analysis of CARD16 in glioma(B), GBM(C) and Low-grade glioma (LGG)(D) samples in TCGA database. The results of OS analysis demonstrated that the CARD16 expression was negatively correlated with overall survival in glioma. E. The expression of CARD16 in glioma increased with the WHO grades in the CGGA database.

Fig.S3: Figure A showed the metastasis related markers E-cadherin, N-cadherin and MMP-2 in the CARD16 knockdown LN18 and T98G cells and the controls. Figure B revealed that CARD16 knockdown had no downregulation effect on CASP1, ASC, and Akt pathway. Figure C: The mRNA expression comparison of CARD-containing genes between CARD16 knockdown LN18 and control. Figure D: The mRNA expression comparison of Akt1 and PIK3CA

between CARD16 knockdown LN18 and control.

Fig.S3: Figure A showed that the GSEA enrichment plot of the FOXO-meditated transcription(left), FOXO-meditated transcription of cell death genes(middle), and Death Receptor Signaling(right) were upregulated in the CARD16 knockdown LN18 cells. Figure B revealed that CARD16 was closely related to central nervous system tumor and malignant glioma. Figure C: The expression comparison of FOXO1 in normal brain samples in the GTEx and glioma samples in the CGGA database. D and E. Western blotting(B) and immunohistochemical(C) assays of FOXO1 in the paired peri-tumor and tumor tissues in GBM specimen. F. exhibited the ideograph of TFBS on TRAIL promoter. Fig. G showed binding sequence logos of FOXO1 in the JASPAR database.

Fig.S4 A and B. Flow cytometry analysis with Annexin V-PI double staining revealed that silencing of FOXO1 reduced the percentage of apoptotic cells under CARD16 knockdown conditions in LN18(A) and T98G(B).

Fig.S5: A: DEGs analysis showed that P21/CDKN1A was remarkably enhanced in CARD16 knockdown LN18 cells. B: GSEA analysis revealed that CDK-mediated phosphorylation was enriched in LN18 NC cells. C: The qPCR assays of P21 and CDK2 showed that P21 was upregulated in CARD16 knockdown GBM cells, while CDK2 mRNA level was not changed.

Supplementary Table

Supplementary Table S1: sheet A: Patients Data of one hundred glioma specimen slides. sheet B: Patients Data of forty fresh paired GBM and peri-tumor tissue specimens.

Supplementary Table S2: Differential expression genes list in the RNA-seq of LN18 NC and LN18 CARD16-sh cells.
