# Supplementary figures and images for: CARD16 restores tumorigenesis and restraints apoptosis in glioma cells Via FOXO1/TRAIL axis
Source: Cell Death Dis. 2024 Nov 8;15(11):804. doi: 10.1038/s41419-024-07196-2 (PMC11549220; doi:10.1038/s41419-024-07196-2)

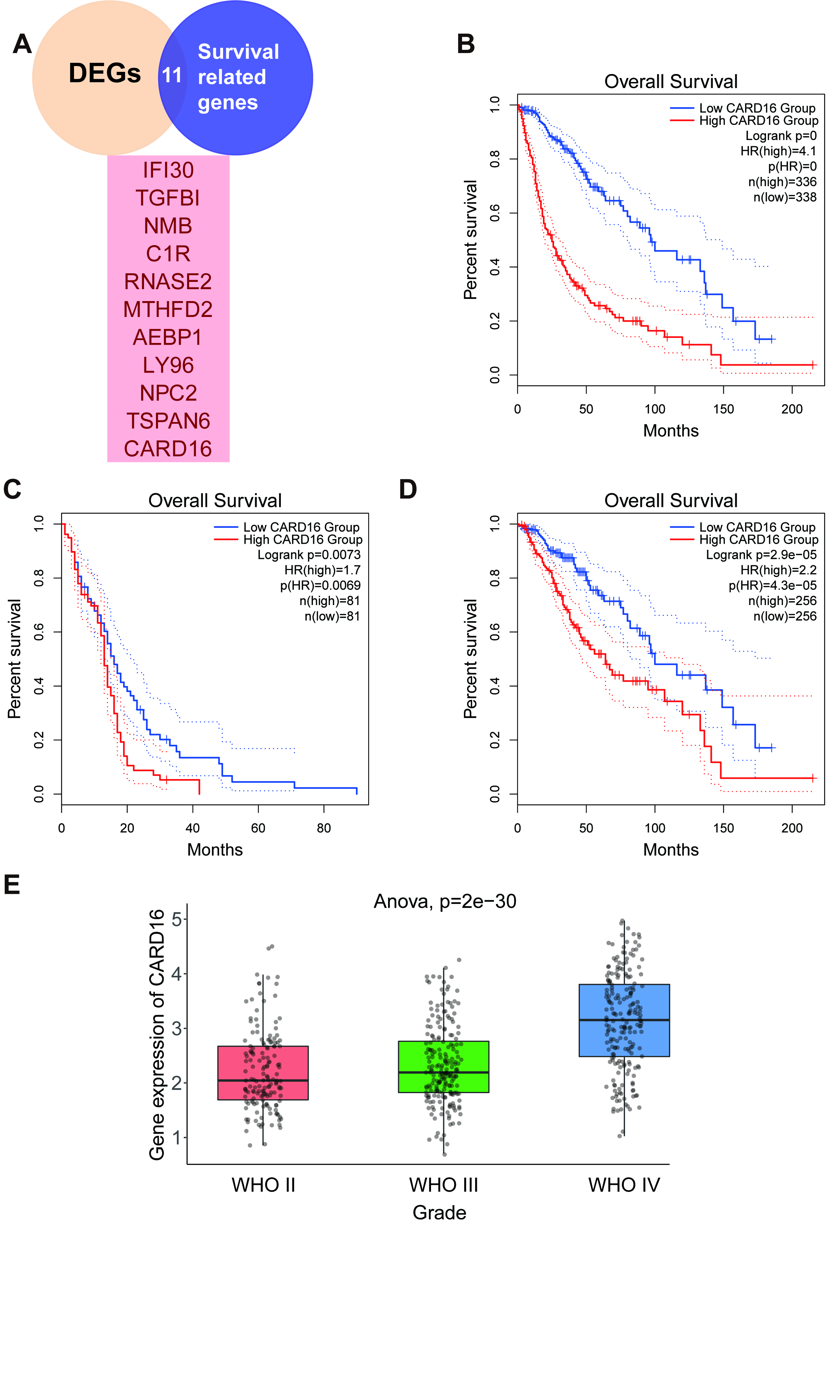

Supplement: Supplementary file 2 — Supplementary Figure. S1 [file 41419_2024_7196_MOESM2_ESM.tif]

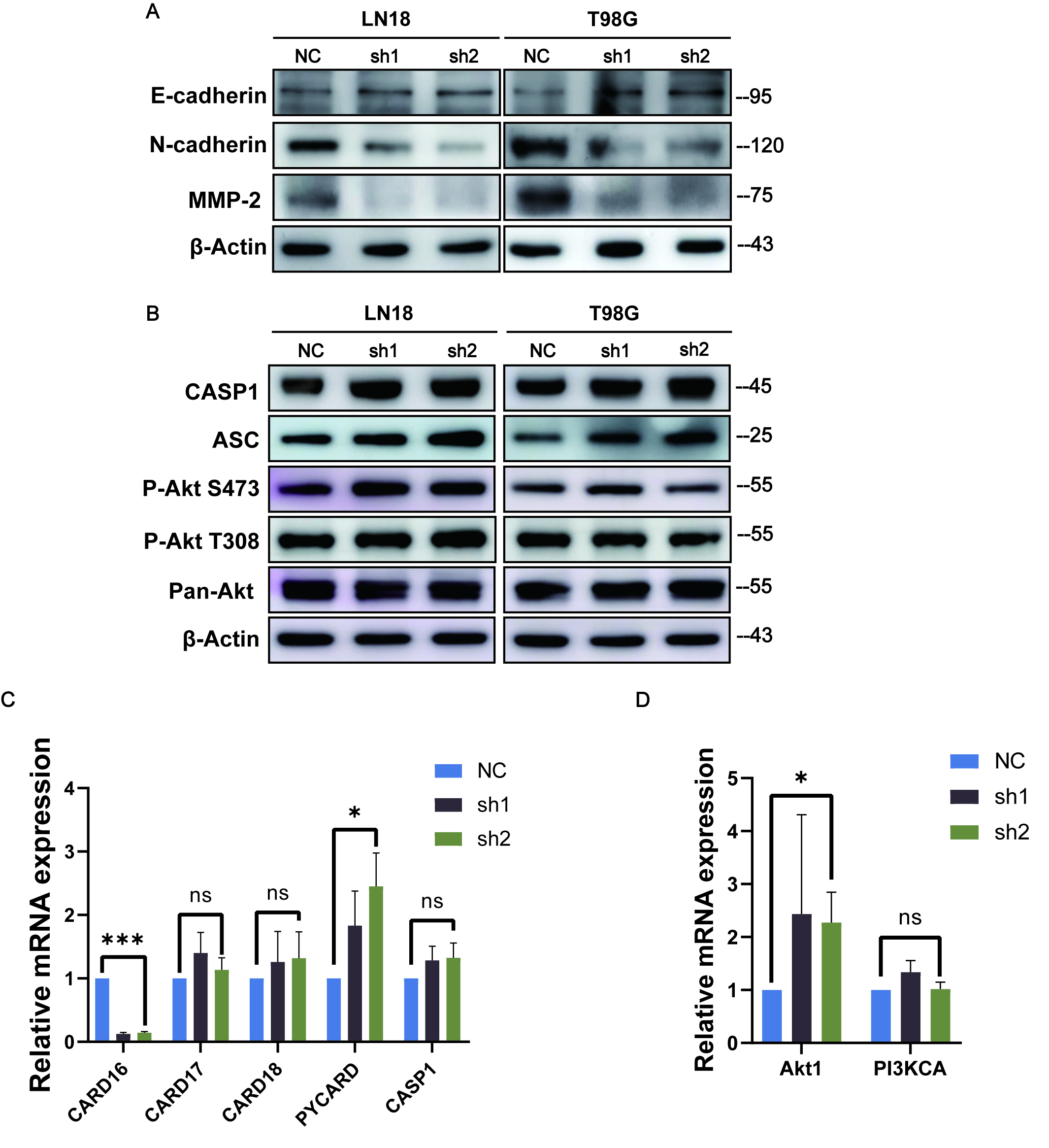

Supplement: Supplementary file 3 — Supplementary Figure. S2 [file 41419_2024_7196_MOESM3_ESM.tif]

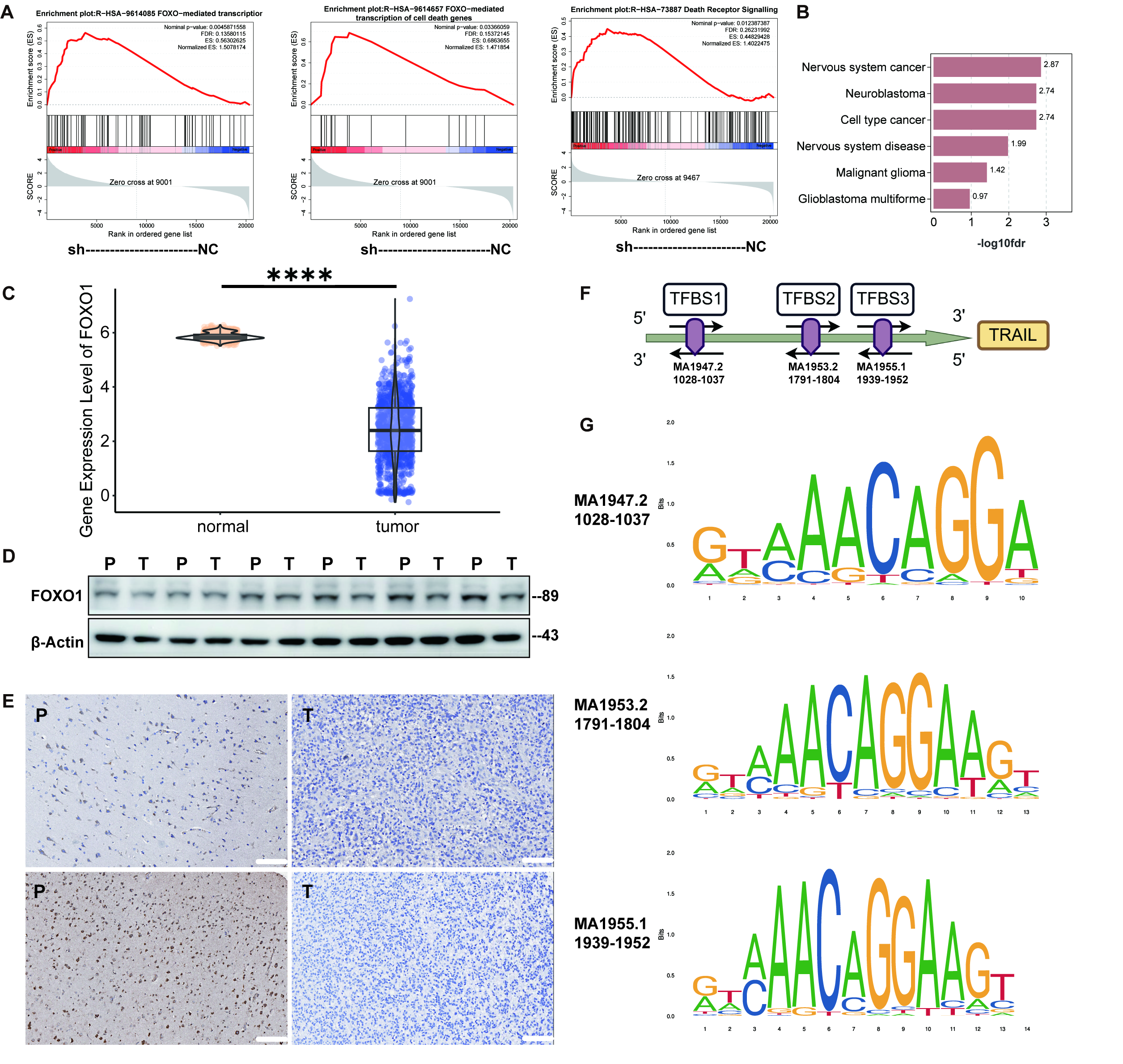

Supplement: Supplementary file 4 — Supplementary Figure. S3 [file 41419_2024_7196_MOESM4_ESM.tif]

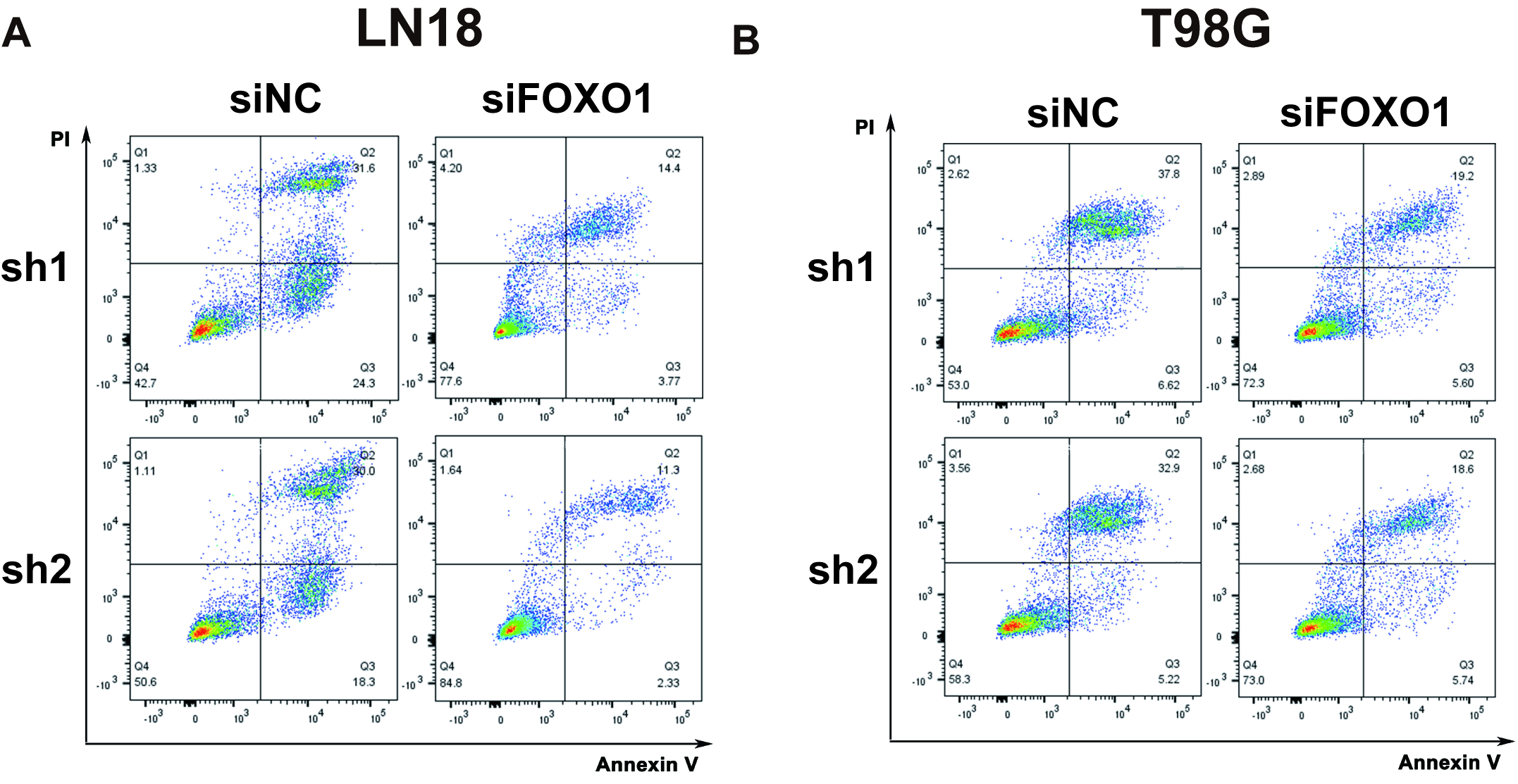

Supplement: Supplementary file 5 — Supplementary Figure. S4 [file 41419_2024_7196_MOESM5_ESM.tif]

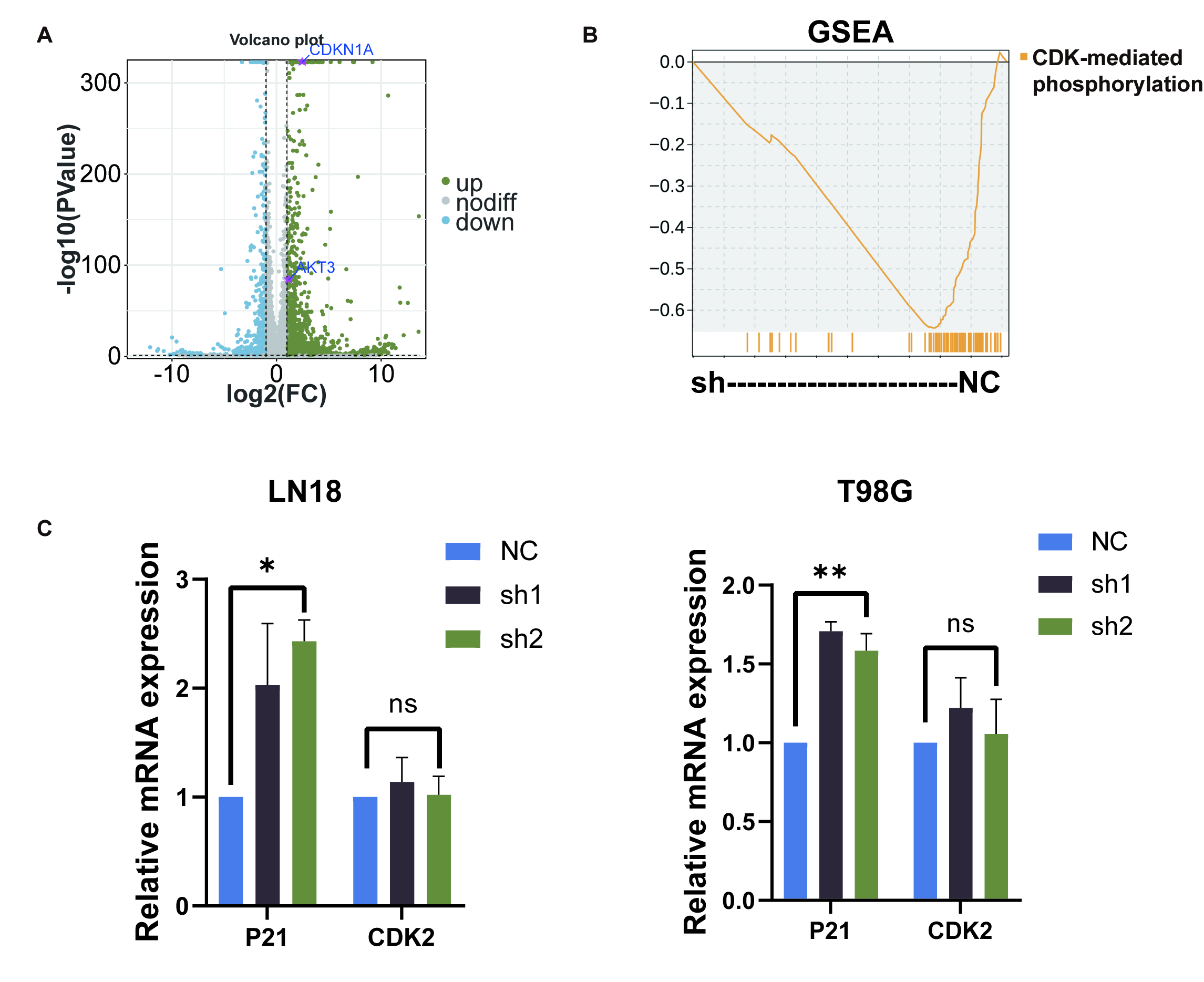

Supplement: Supplementary file 6 — Supplementary Figure. S5 [file 41419_2024_7196_MOESM6_ESM.tif]
