# Supplementary material for: CARD16 restores tumorigenesis and restraints apoptosis in glioma cells Via FOXO1/TRAIL axis
Source: Cell Death Dis. 2024 Nov 8;15(11):804. doi: 10.1038/s41419-024-07196-2 (PMC11549220; doi:10.1038/s41419-024-07196-2)

Figure 1D

CARD16-1

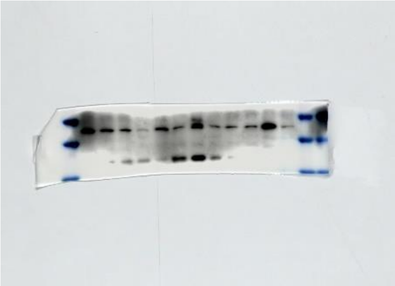

Actin-1

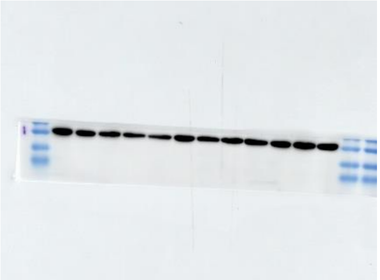

CARD16-2

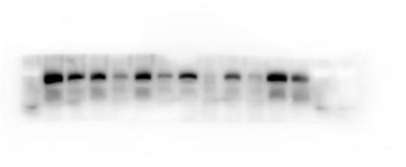

Actin-2

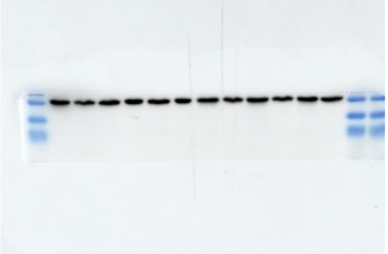

CARD16-3

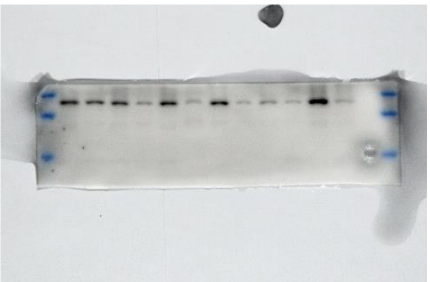

Actin-3

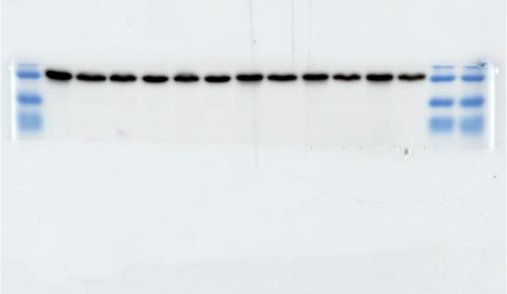

Figure 1H

CARD16

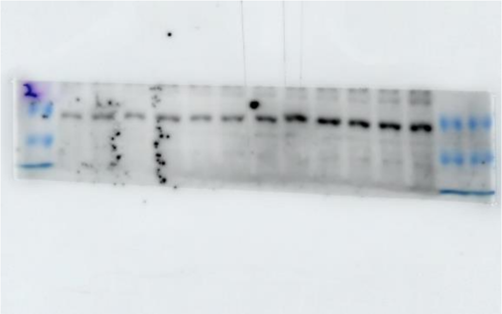

Actin

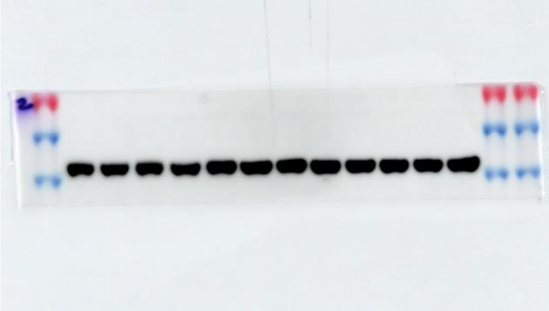

Figure 2A

CARD16 Actin

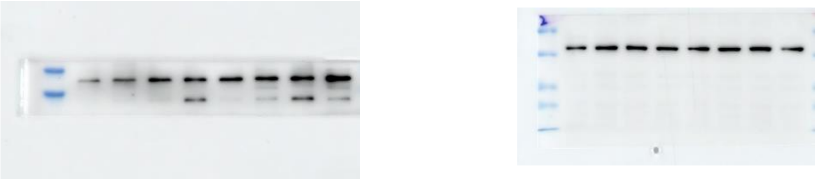

Figure 2C

CARD16 LN18 T98G HS683

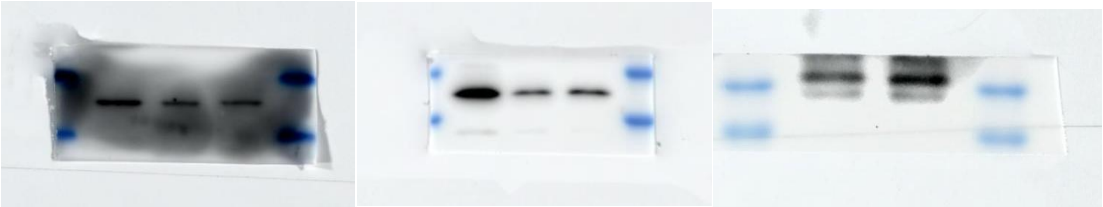

β-Actin LN18 T98G HS683

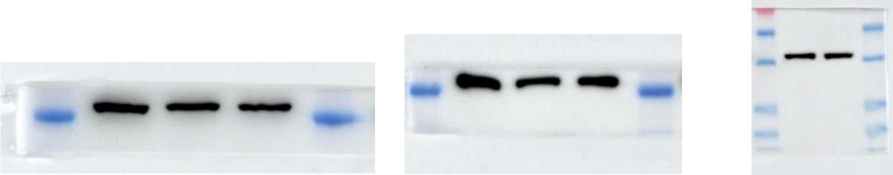

Figure 2F

P65 LN18 T98G HS683

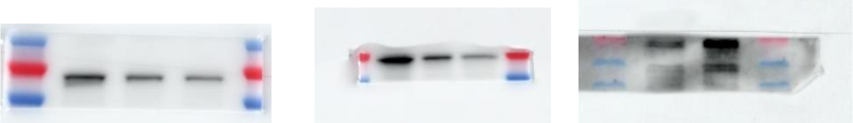

VEGFA LN18 T98G HS683

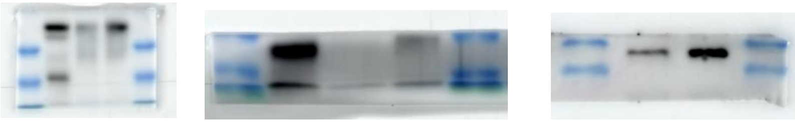

Cyclin E1 LN18 T98G HS683

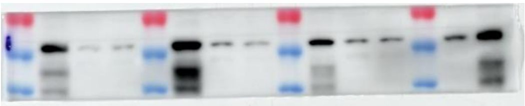

Cyclin D1 LN18 T98G HS683

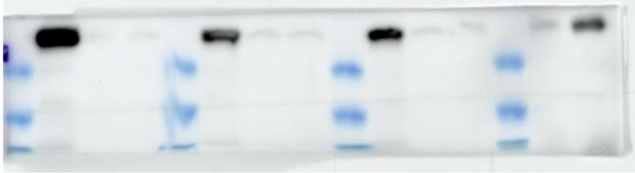

β-actin LN18 T98G HS683

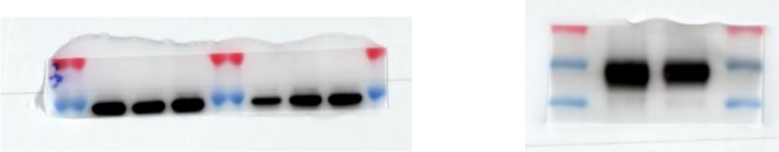

Figure 3A

Cleaved PARP LN18 T98G

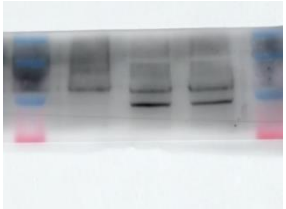

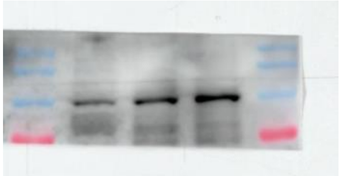

Caspase8 LN18 T98G

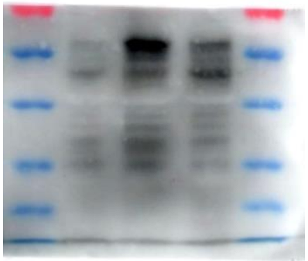

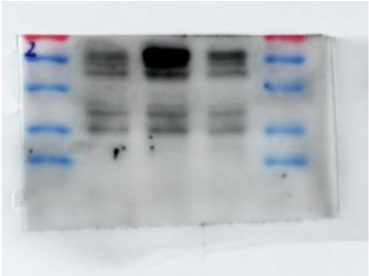

Caspase9 LN18 T98G

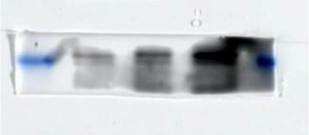

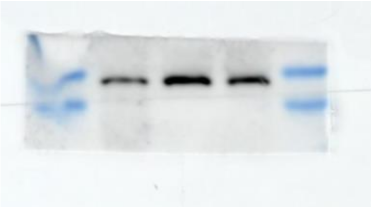

Cleaved Caspase3 LN18 T98G

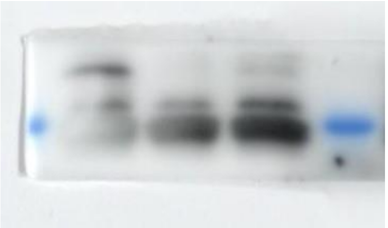

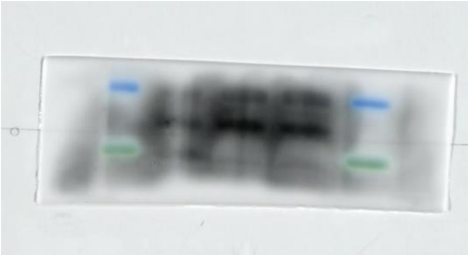

β-actin LN18 T98G

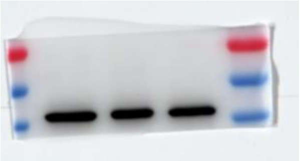

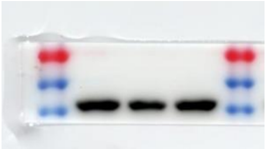

Figure 5D

FOXO1 LN18 T98G

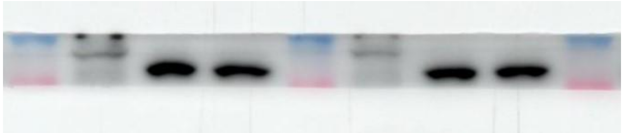

p-FOXO1S249 LN18 T98G

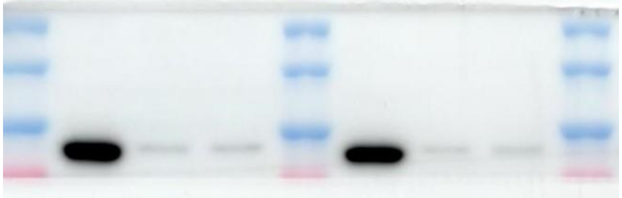

Caspase8 LN18 T98G

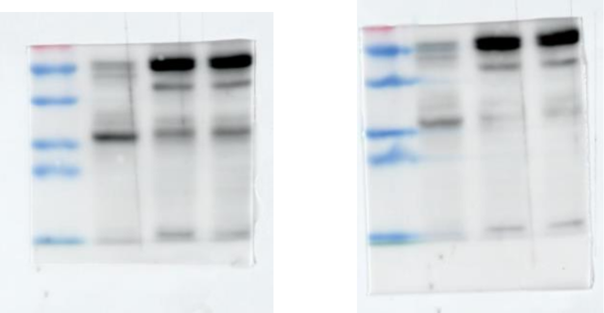

DR5 LN18 T98G

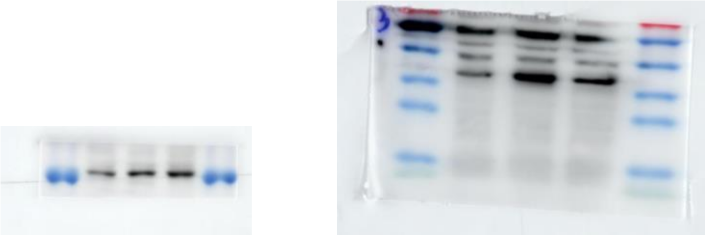

TRAIL LN18 T98G

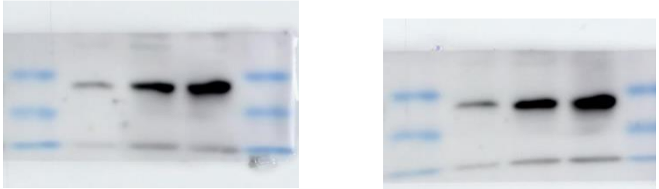

Tubulin LN18 T98G

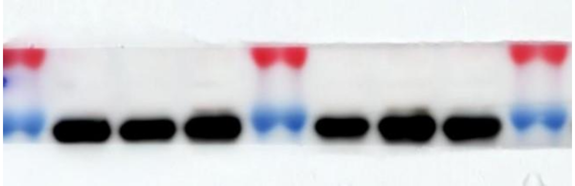

Figure 5J

FOXO1 LN18 T98G

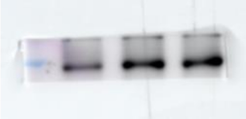

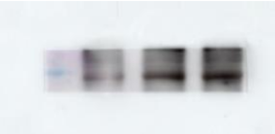

p-FOXO1S249 LN18 T98G

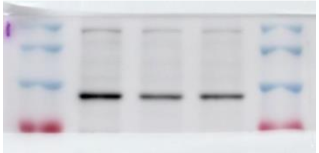

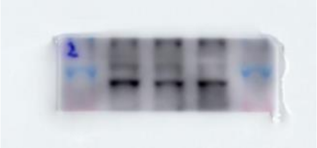

Figure 5D

Lamin B1 LN18 T98G

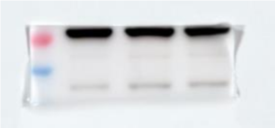

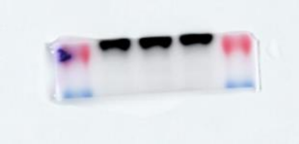

Figure 6A

FOXO1 LN18 T98G

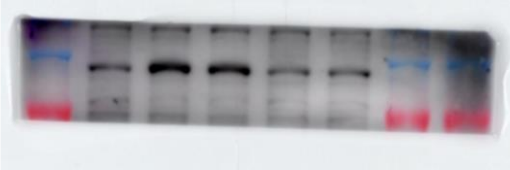

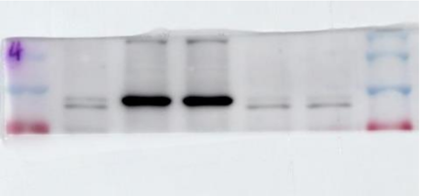

Caspase8 LN18 T98G

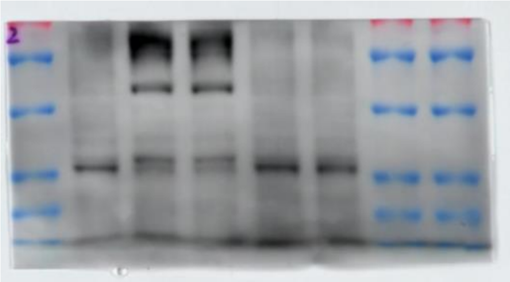

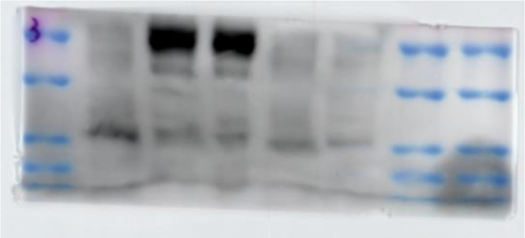

TRAIL LN18 T98G

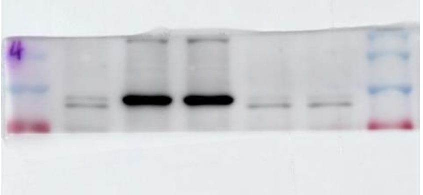

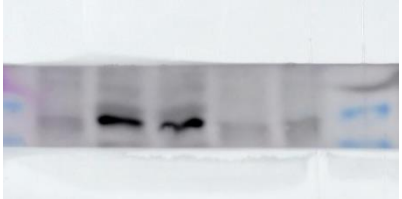

β-actin LN18 T98G

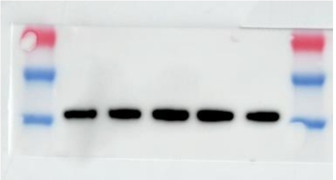

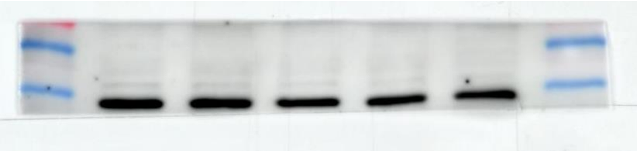

Figure 8A

FOXO1 LN18 T98G

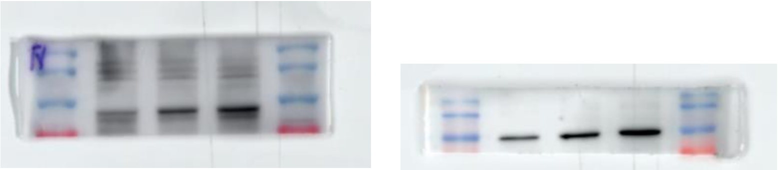

p-FOXO1ser256 LN18 T98G

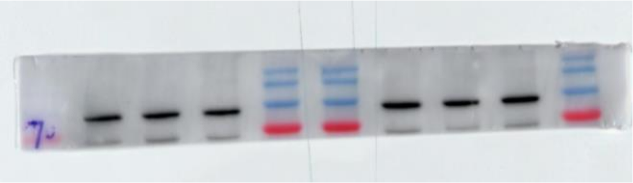

p-FOXO1ser249 LN18 T98G

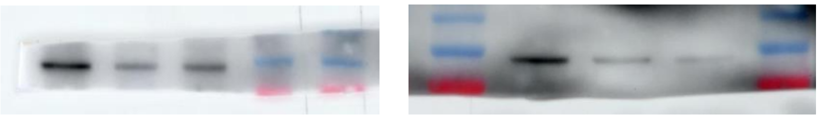

p-CDK2Thr160 LN18 T98G

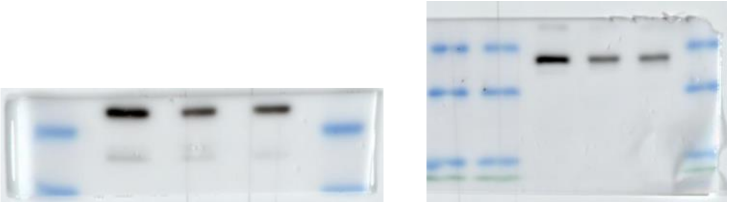

CDK2 LN18 T98G

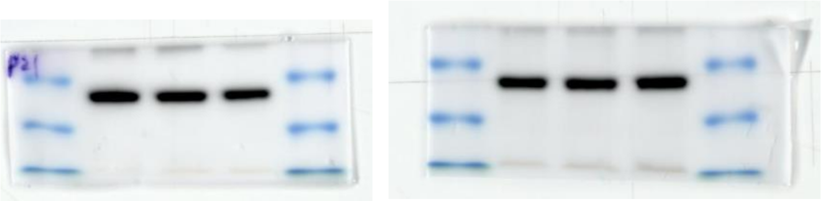

P21 LN18 T98G

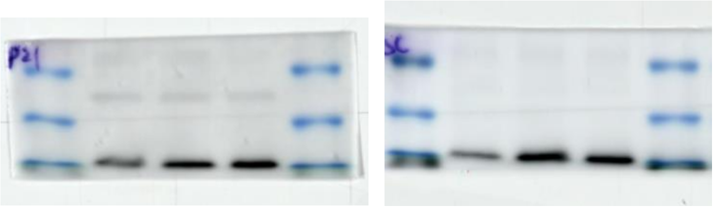

β-Actin LN18 T98G

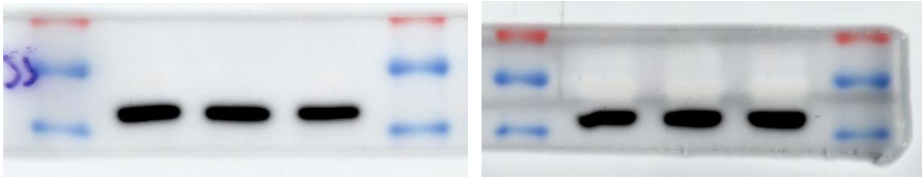

Figure 8B

IP Ubiquitin LN18 T98G

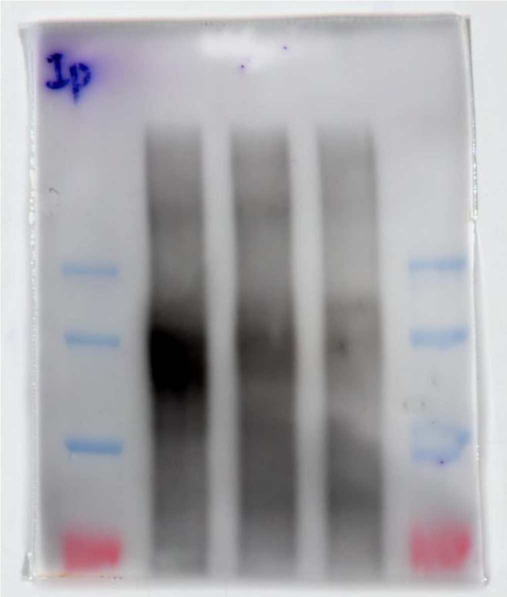

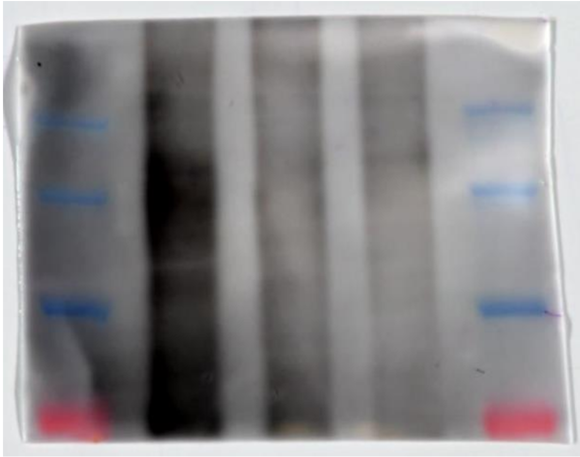

IP CDK2 LN18 T98G

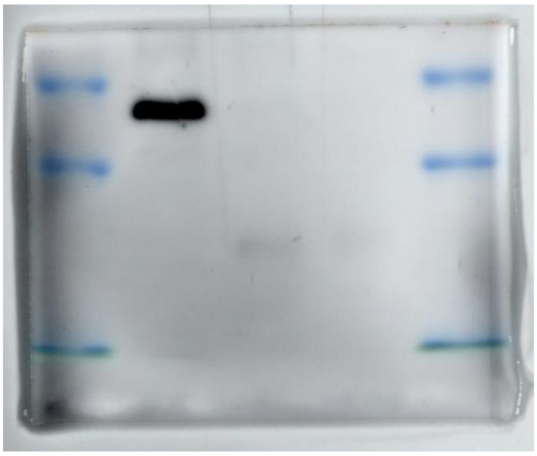

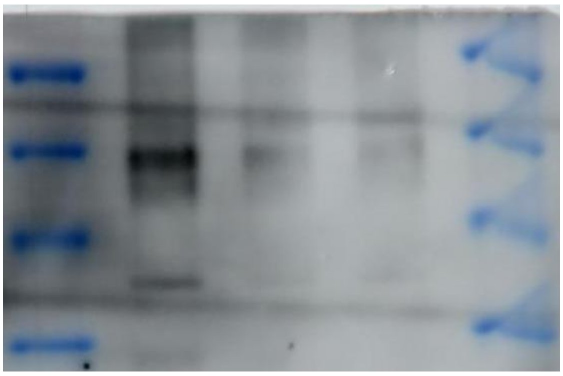

Input p-FOXO1ser249 LN18 T98G

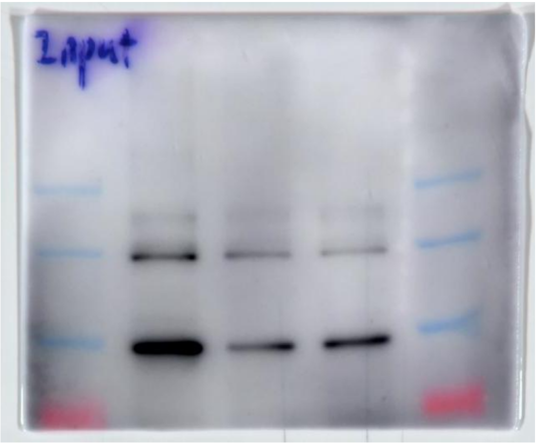

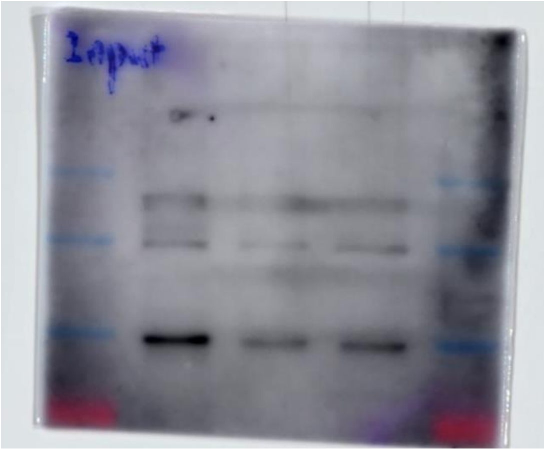

Input CDK2 LN18 T98G

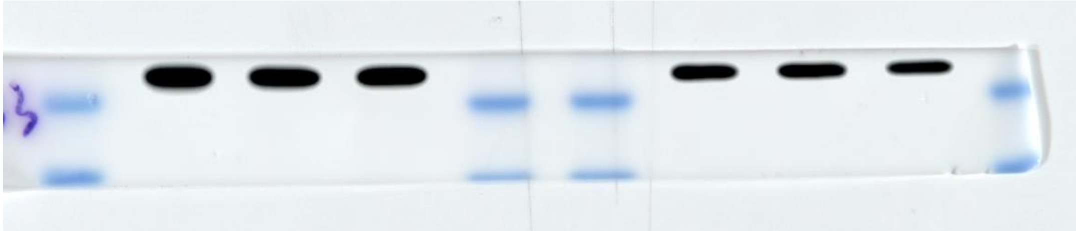

Input p-CDK2Thr160 LN18 T98G

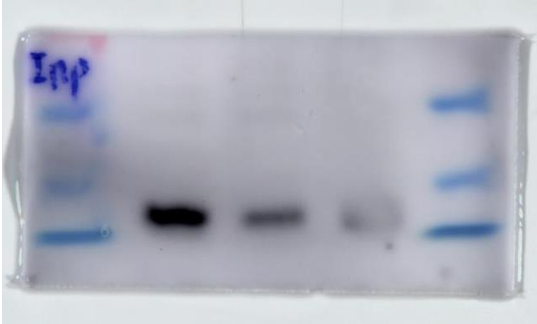

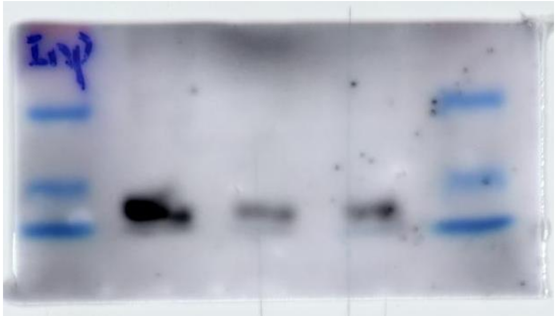

Input p21 LN18 T98G

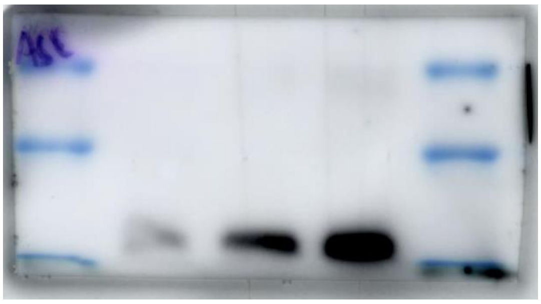

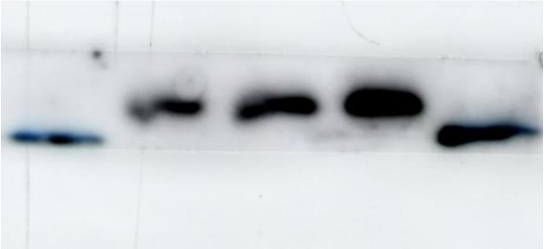

Input Tunulin LN18 T98G

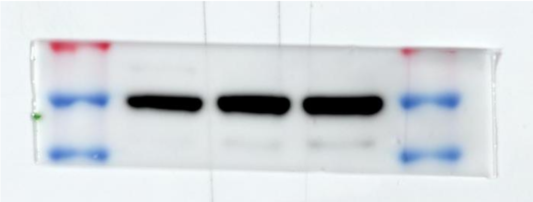

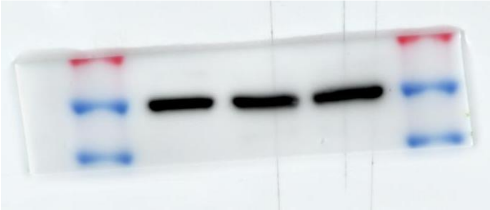

Fig 8C P21 LN18 sh1 LN18 sh2

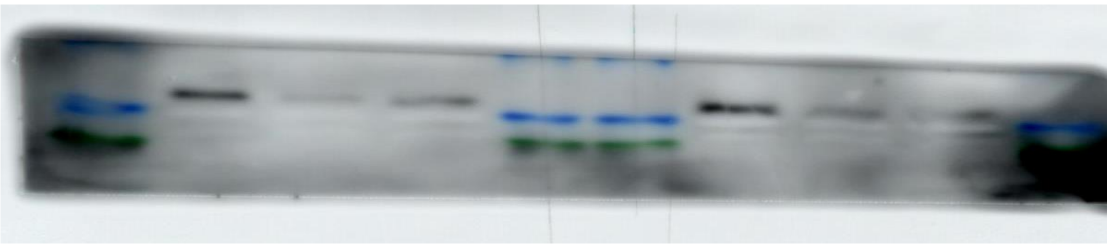

Fig 8C Tubulin LN18sh1 LN18 sh2

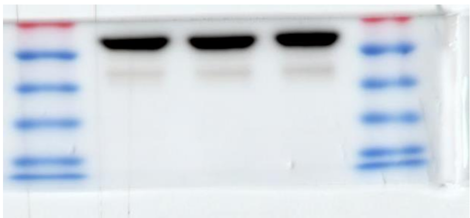

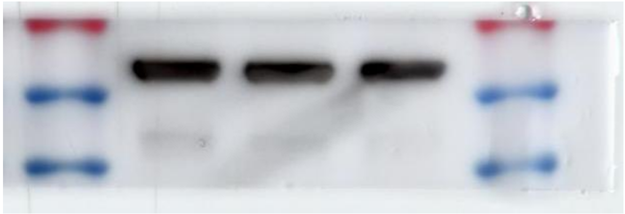

Figure 8D

IP Ubiquitin LN18

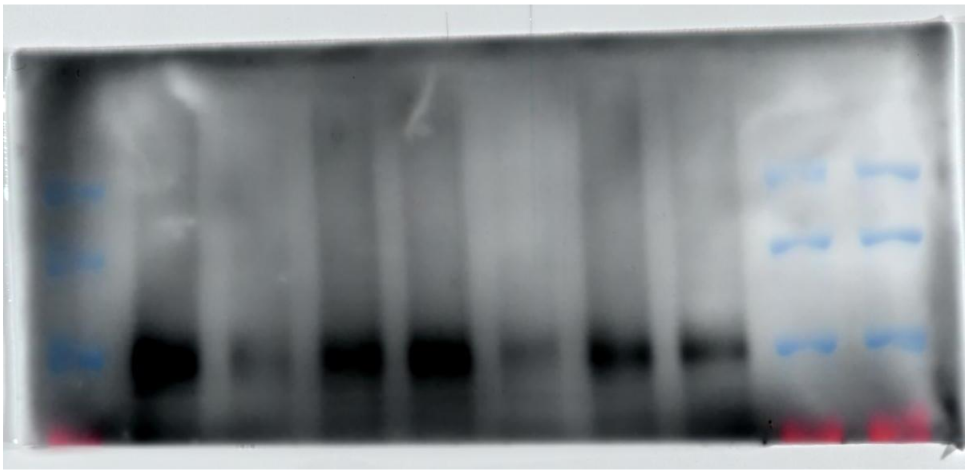

IP Ubiquitin T98G

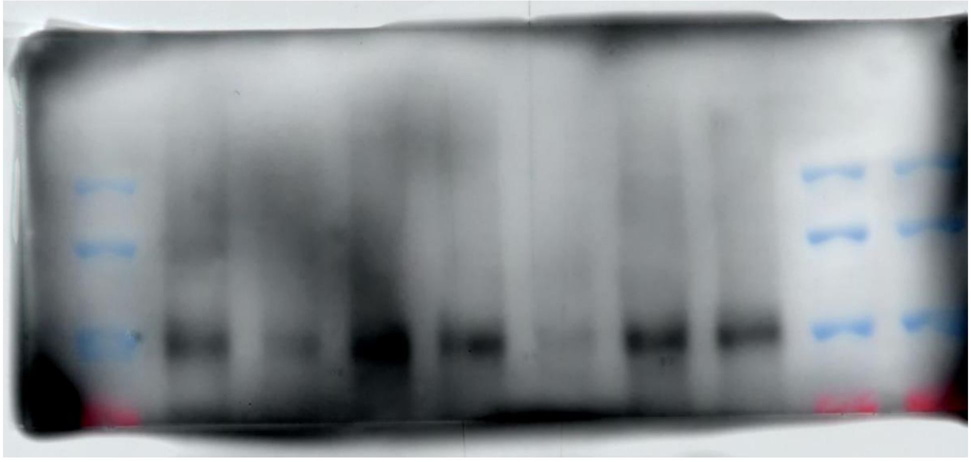

IP CDK2 LN18

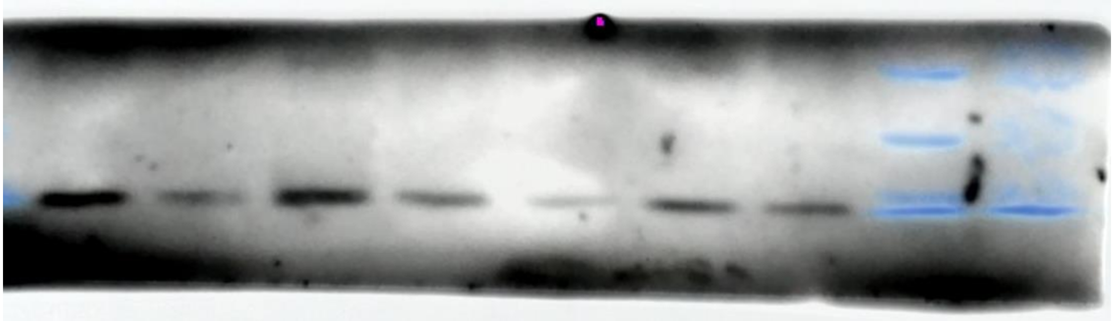

IP CDK2 T98G

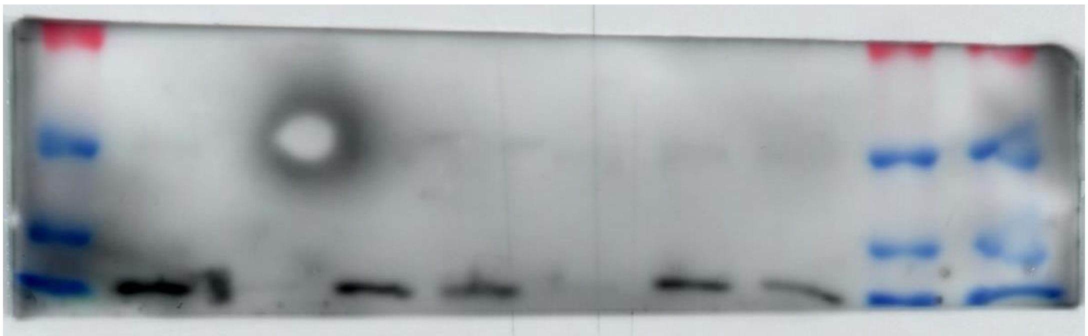

input p-FOXO1 Ser249 LN18

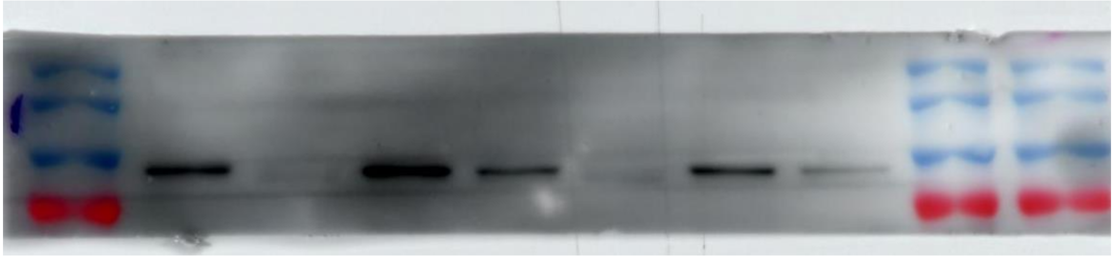

input p-FOXO1 Ser249 T98G

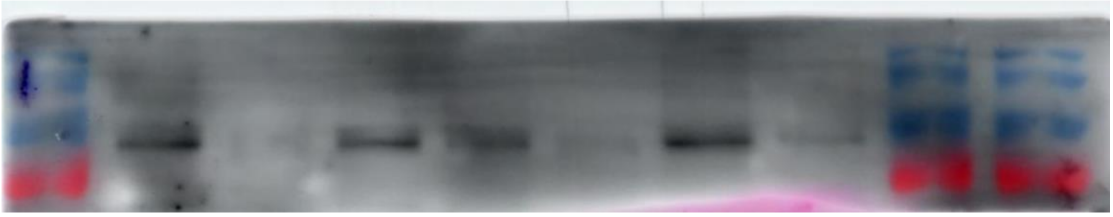

input FOXO1 LN18

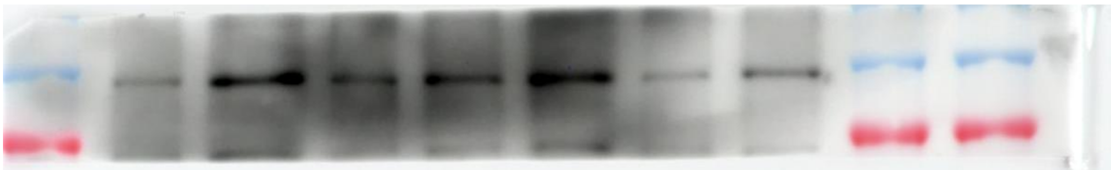

input FOXO1 T98G

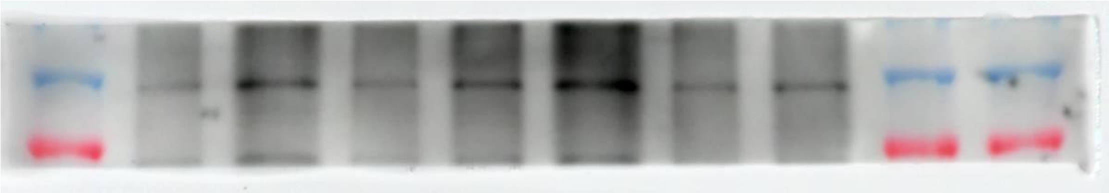

input CDK2 LN18

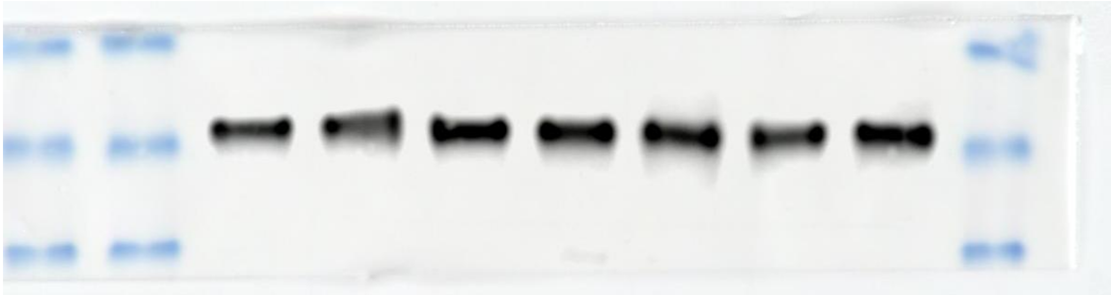

input CDK2 T98G

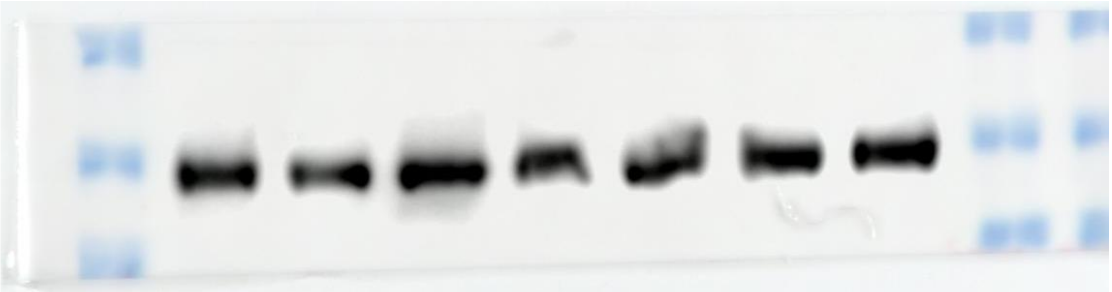

input p-CDK2 Thr160 LN18

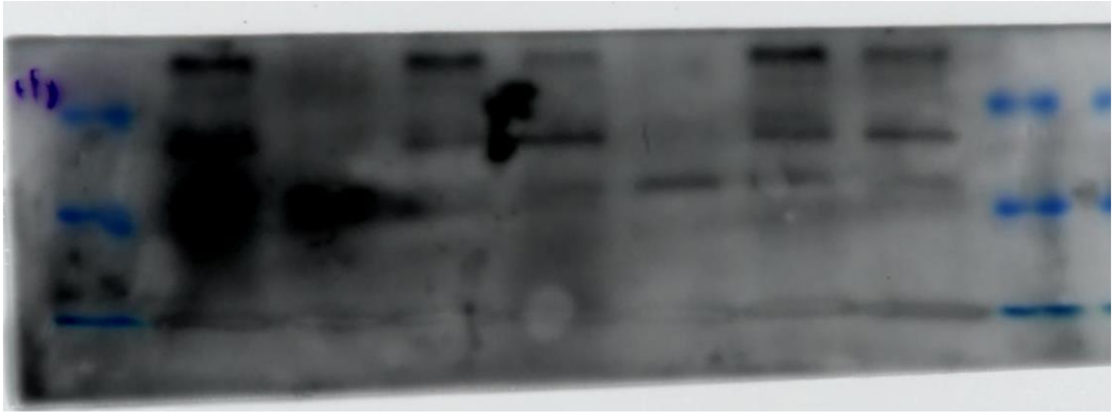

input p-CDK2 Thr160 T98G

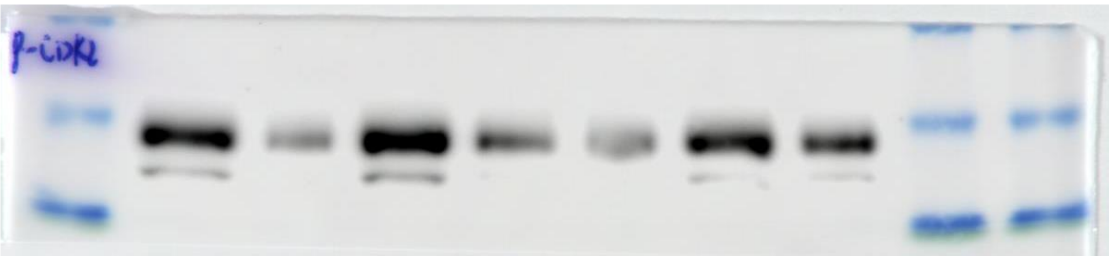

input p21 LN18

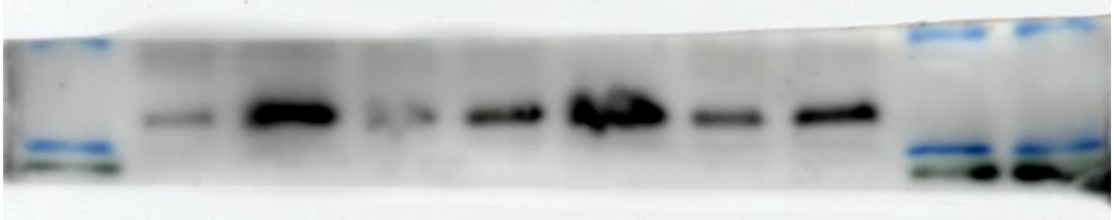

input p21 T98G

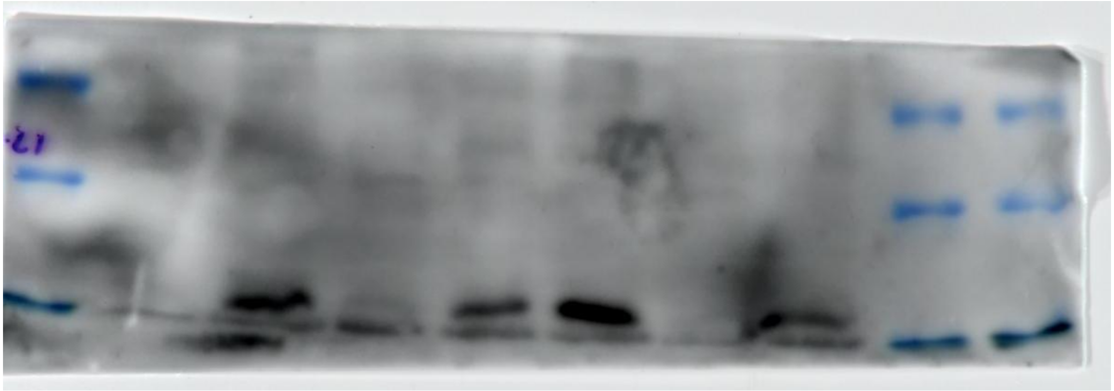

Input Tubulin LN18

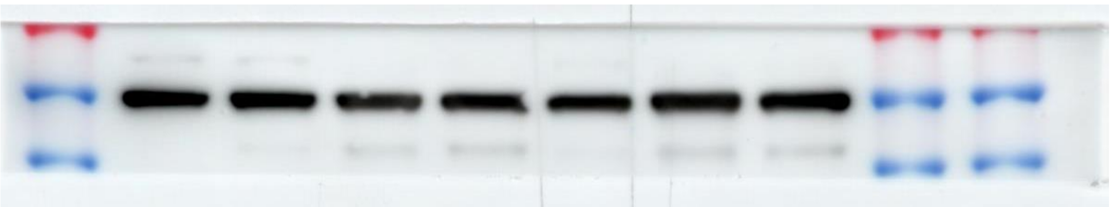

Input Tubulin T98G

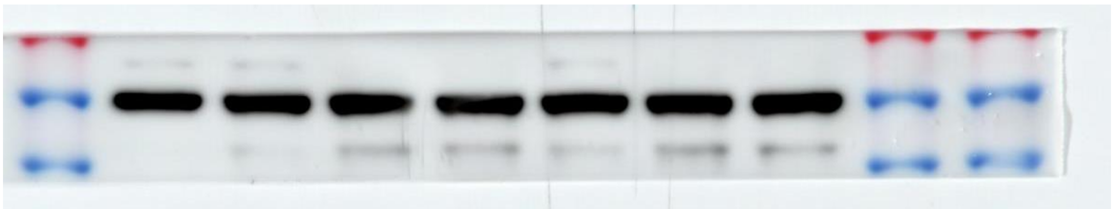

Supplementary Fig S2A

E-cadherin LN18 T98G

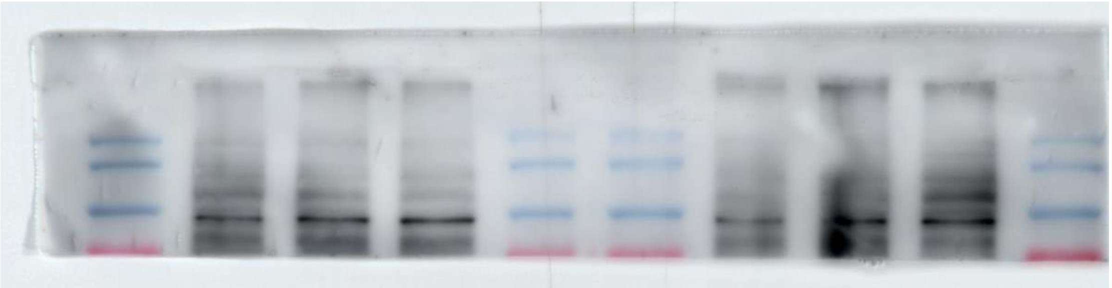

N-cadherin LN18 T98G

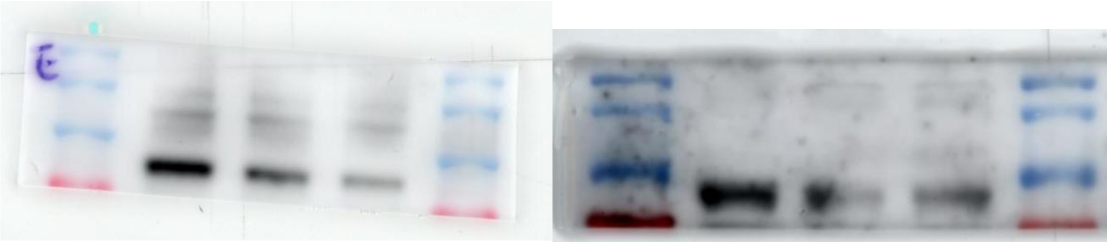

MMP-2 LN18 T98G

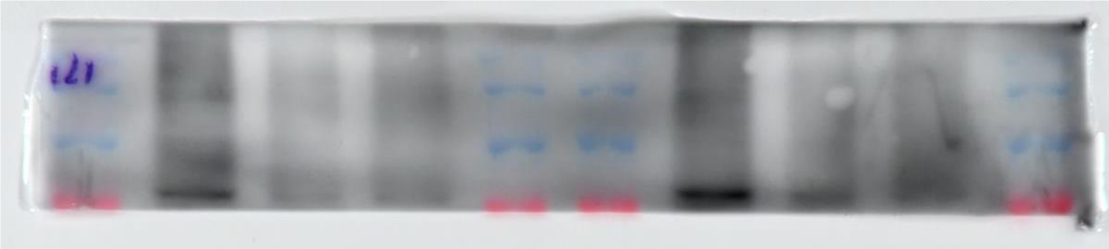

β-actin LN18 T98G

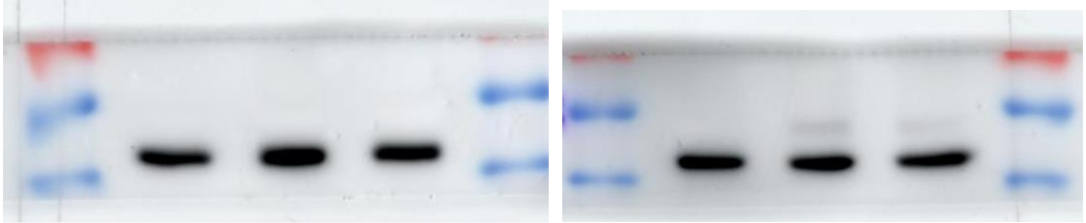

Supplementary Fig S2B

PYCARD LN18 T98G

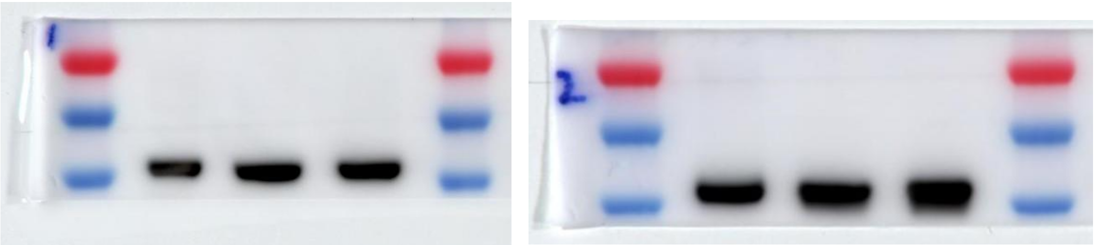

PYCARD LN18 T98G

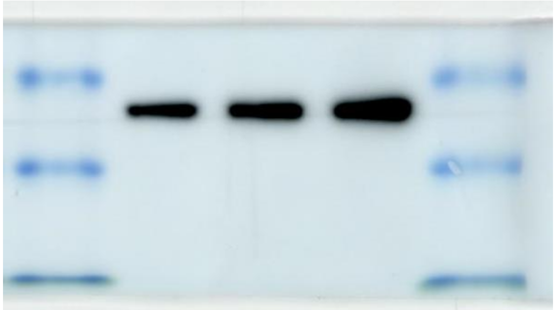

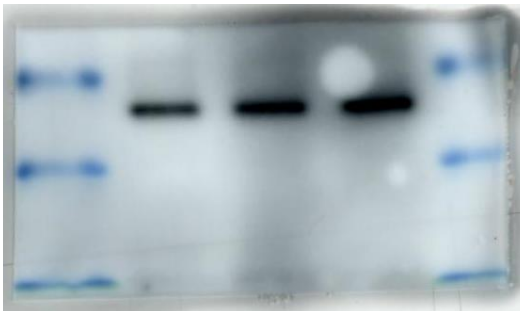

P-AKT Ser473 LN18 T98G

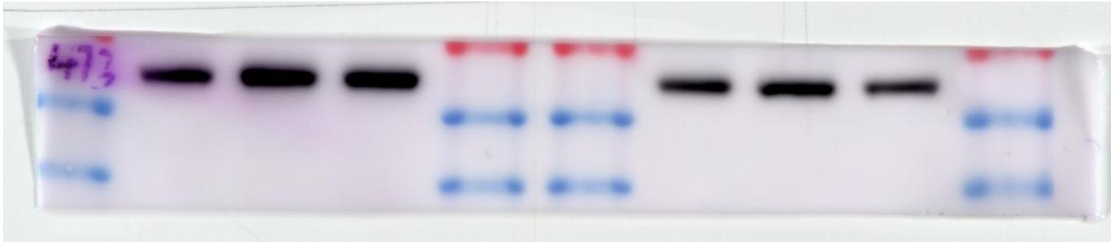

P-AKT Thr308 LN18 T98G

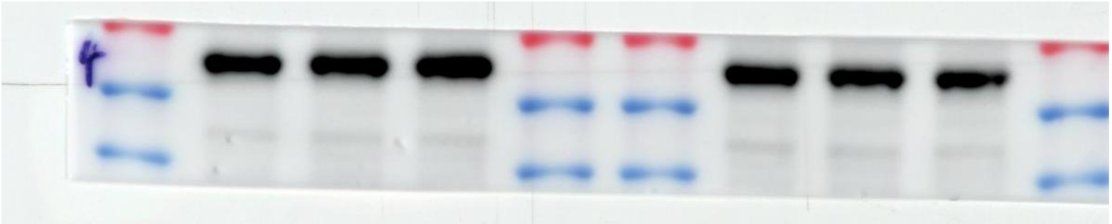

Pan-AKT LN18 T98G

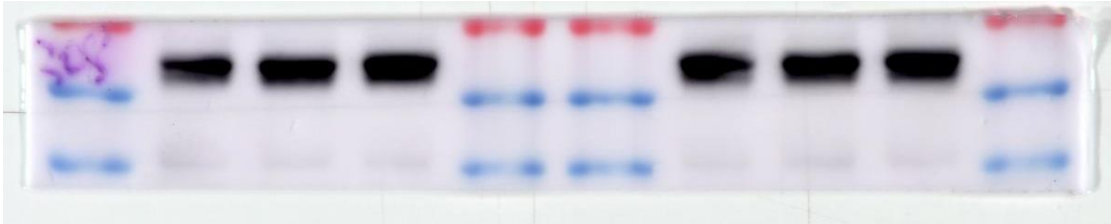

β-actin LN18 T98G

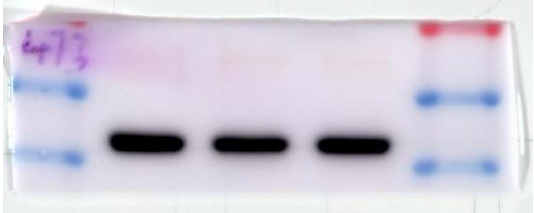

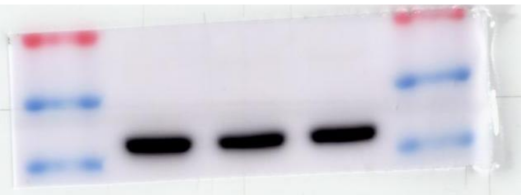

Supplementary Fig S3D

FOXO1

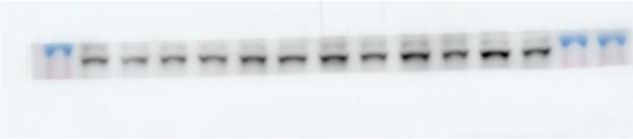

β-actin

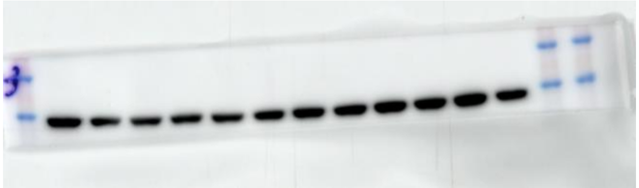

Supplement: Supplementary file 9 — Supplementary Original WB [file 41419_2024_7196_MOESM9_ESM.pdf]
